# Genome flux and stasis in a five millennium transect of European prehistory

**DOI:** 10.1038/ncomms6257

**Published:** 2014-10-21

**Authors:** Cristina Gamba, Eppie R. Jones, Matthew D. Teasdale, Russell L. McLaughlin, Gloria Gonzalez-Fortes, Valeria Mattiangeli, László Domboróczki, Ivett Kővári, Ildikó Pap, Alexandra Anders, Alasdair Whittle, János Dani, Pál Raczky, Thomas F. G. Higham, Michael Hofreiter, Daniel G Bradley, Ron Pinhasi

**Affiliations:** 1 School of Archaeology, University College Dublin, Belfield, Dublin 4, Ireland; 2 Conway Institute, University College Dublin, Belfield, Dublin 4, Ireland; 3 Smurfit Institute of Genetics, Trinity College Dublin, Dublin, Ireland; 4 Institute of Biochemistry and Biology, University of Potsdam, Karl-Liebknecht-Straße 24-25, 14476 Potsdam, Germany; 5 Dobó István Castle Museum, Vár utca 1, H-3300 Eger, Hungary; 6 JPAC-Central Identification Laboratory, 310 Worchester Avenue, Building. 45 Joint Base Pearl Harbor-Hickam, Honalulu, Hawaii 96853-5530, USA; 7 Department of Anthropology, Hungarian Natural History Museum, Ludovika tér 2-6, 1083 Budapest, Hungary; 8 Eötvös Loránd University, Faculty of Humanities, Institute of Archaeological Sciences, Múzeum körút 4/b, H-1o88 Budapest, Hungary; 9 Department of Archaeology and Conservation, Cardiff University, Cardiff CF10 3EU, UK; 10 Déri Museum, Déri tér 1, H-4026 Debrecen, Hungary; 11 Oxford Radiocarbon Accelerator Unit, Research Laboratory for Archaeology and the History of Art, University of Oxford, Dyson Perrins Building, South Parks Road, OX1 3QY Oxford, UK; 12 Earth Institute, University College Dublin, Belfield, Dublin 4, Ireland

## Abstract

The Great Hungarian Plain was a crossroads of cultural transformations that have shaped European prehistory. Here we analyse a 5,000-year transect of human genomes, sampled from petrous bones giving consistently excellent endogenous DNA yields, from 13 Hungarian Neolithic, Copper, Bronze and Iron Age burials including two to high (~22 × ) and seven to ~1 × coverage, to investigate the impact of these on Europe’s genetic landscape. These data suggest genomic shifts with the advent of the Neolithic, Bronze and Iron Ages, with interleaved periods of genome stability. The earliest Neolithic context genome shows a European hunter-gatherer genetic signature and a restricted ancestral population size, suggesting direct contact between cultures after the arrival of the first farmers into Europe. The latest, Iron Age, sample reveals an eastern genomic influence concordant with introduced Steppe burial rites. We observe transition towards lighter pigmentation and surprisingly, no Neolithic presence of lactase persistence.

## Abstract (web summary)

Recent advances in high-throughput sequencing techniques have enabled the analysis of ancient human genomes. Here the authors sequence ancient human genomes that span a period of 5,000 years, to understand the ancestral influence on Europe's genetic landscape.

## Graphical abstract

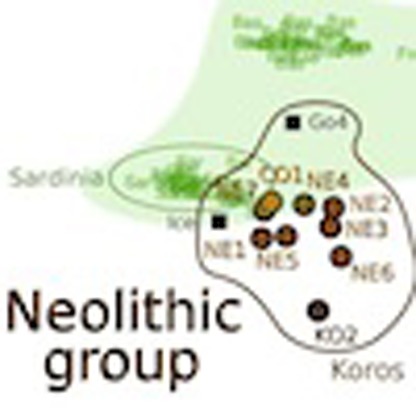

Thanks to the development of high-throughput sequencing techniques within the last decade, ancient human genomes have become accessible and now form an exciting resource that allows the testing of archaeological hypotheses *in situ*. However, sample preservation still represents a substantial challenge, particularly the typically low fraction of endogenous DNA within the overall recovered sequence data^1^,^2^,^3^.

The potential of ancient genomes to shed new light on European human prehistory is illustrated by those individuals whose complete or partial autosomal genomes have been determined to date^1^,^2^,^4^,^5^,^6^,^7^,^8^. Nineteen of these samples are hunter-gatherers, while only two complete and four partial ancient farmers’ genomes (from Tyrol, Germany and Sweden) have been sequenced to date^2^,^5^,^6^,^8^.

Although no diachronic series has yet investigated temporal genome-wide dynamics within a defined European region, in prior analyses hunter-gatherers’ genomes have fallen outside the range of modern European variation, while farmers’ samples showed an affinity to Southern Europeans, particularly present-day Sardinians.

The Great Hungarian Plain, situated between Mediterranean and temperate Europe, was throughout prehistory a place of cultural and technological transformations as well as a major meeting point of Eastern and Western European cultures^9^. Farming began in this region with the Early Neolithic Körös culture, 6,000–5,500 cal BC, which is part of the Early Neolithic of Southeast Europe^10^,^11^,^12^, followed ~5,500 cal BC by the Middle Neolithic Linearbandkeramik (LBK) culture that consisted of two synchronous regional groups: the Alföld Linear Pottery (ALP, also Bükk) culture^13^,^14^ and the Transdanubian LBK variant in West Hungary^15^, which later dispersed agriculture into Central Europe and became the dominant farming culture of Europe. Locally, it developed into the Late Neolithic (*ca.* 5,000–4,500 cal BC) Lengyel culture.

In the Great Hungarian Plain, there is continuity in material culture and settlements between the Late Neolithic and the Copper Age Baden Culture. However, during the Early Bronze Age (2,800–1,800 cal BC), growing demand for metal ores throughout Europe gave rise to new pan-European and intercontinental trading networks^16^. The Early Bronze Age cultures of the Great Hungarian Plain incorporated technology, settlement type and material cultural elements from the contemporaneous Bronze Age cultures of the Near East, Steppe and Central Europe. Finally, during the early phase of the Iron Age (first millennium BC), a variant of the Central European Hallstatt culture inhabited Transdanubia, whereas pre-Scythian (‘Mezőcsát communities’ of unknown origin) and later Scythian cultures prevailed further East on the Great Hungarian Plain.

A compelling question is whether these major prehistoric transitions involved exogenous population influxes. Particularly, in the transition to agriculture in this gateway of the European Neolithic, what level of interaction and intermarriage may have occurred between local hunter-gatherer and non-local farmers? Archaeological evidence for the presence of Mesolithic hunter-gatherers in Southeast Europe is limited to a few small regions^9^ while a greater Mesolithic presence can be documented for parts of Northern Hungary and further northwards.

Here we assess the imprint of this series of major cultural and technological shifts on the genomes of Central European prehistory through the analysis of a 5,000-year temporal transect of complete and partial genomes of individuals from archaeological sites in the Great Hungarian Plain.

## Results

### The petrous bone and differential DNA yields

**Figure 1:**
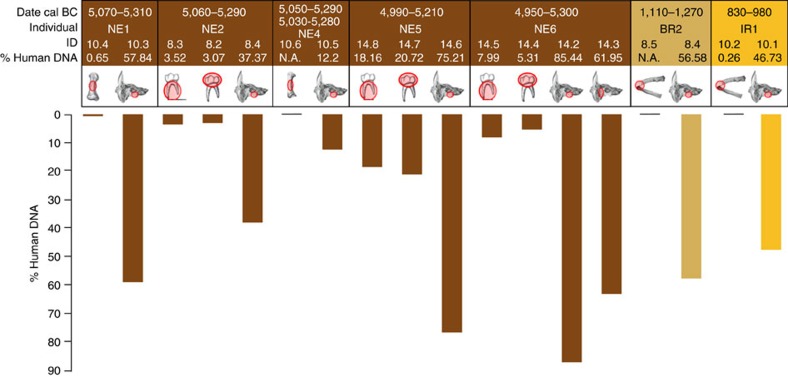
Petrous bones versus non-petrous bones. Percentage of non-clonal endogenous DNA recovered after shotgun sequencing. The sampled bone/tooth portion is circled in red. N.A. indicates that the library did not pass quality assessment for sequencing.

Although the advantages of genome-wide analysis are numerous, such data have not been routinely accessible due to the typically low endogenous DNA content in human bones in most archaeological contexts^1^,^2^,^3^. We compared endogenous DNA content from the petrous portion of the temporal bone, the densest bone in the mammalian body^17^, and paired alternate skeletal elements from six Hungarian skeletons sampled across diverse time depths (Fig. 1 and Supplementary Table 2). The endogenous DNA yields from the petrous samples exceeded those from the teeth by 4- to 16-fold and those from other bones up to 183-fold. Thus, while other skeletal elements yielded human, non-clonal DNA contents ranging from 0.3 to 20.7%, the levels for petrous bones ranged from 37.4 to 85.4% (Fig. 1). We extended this sampling to a further seven petrous bones from Hungary and yields of endogenous DNA remained exceptionally and consistently high (Supplementary Table 3).

### Overall sequencing results and contamination controls

To investigate temporal genome-wide dynamics we sequenced these 13 ancient individuals to one of the three levels of genome coverage: a Neolithic (5,070–5,310 cal BC) and a Bronze Age (1,110–1,270 cal BC) library were sequenced to high coverage (22.1 × and 21.3 × mean coverage, respectively); seven samples were sequenced to ~1 × coverage; and a further four were sequenced to ~0.1 × , resulting in a ~5,000 year genomic transect from the onset of agriculture in this region during the Early Neolithic Körös period to the pre-Scythian Iron Age (Table 1).

We strictly assessed the authenticity of the data. The sequence reads show damage patterns consistent with ancient DNA (Supplementary Fig. 3) and replicate extractions and library preparations in a separate laboratory gave extremely high genotype concordance (>99%) for the two high-coverage genomes (Supplementary Methods). Further, contamination estimates (mean±s.d.) derived from negative controls (0.14%±0.16), mitochondrial DNA (mtDNA; 0.15%±0.24) and X chromosome polymorphisms in males (0.63%±0.31) were consistently low (Supplementary Methods).

### Temporal dynamics of genomic affinity

All analyzed individuals are from defined archaeological contexts from the onset of agriculture in this region during the Early Neolithic Körös period through to the pre-Scythian Iron Age and were directly radiocarbon dated (Table 1). To assess genomic continuity versus change in this time series and to determine how these ancient genomes relate to each other, to other ancient European genomes and to modern-day human populations, we generated a two-dimensional summary of autosomal genomic variation using principal components analysis (PCA) and combined our observed genotype data with published ancient sequences and genotypes of 552 modern individuals from Europe, Caucasus and the Near East^1^,^2^,^6^,^18^,^19^,^20^. Individual plots, similar in resolution to those observed in analyses of full modern single nucleotide polymorphism (SNP) data sets, were subsequently combined into a single plot using Procrustes transformation, following the study by Skoglund *et al.*^2^ This analysis shows clear shifts (including two within multi-phased archaeological sites) in the genomic affinities of the ancient genotypes coinciding with cultural shifts and bracketing a 2,800 year period of Neolithic stasis. Although our sampling is concentrated in the first millennium of this interval, to place a particular emphasis on the Neolithisation process in Southeast/Central Europe, it was constructed to include material from the diverse archaeological phases within the Hungarian Neolithic.

**Figure 2:**
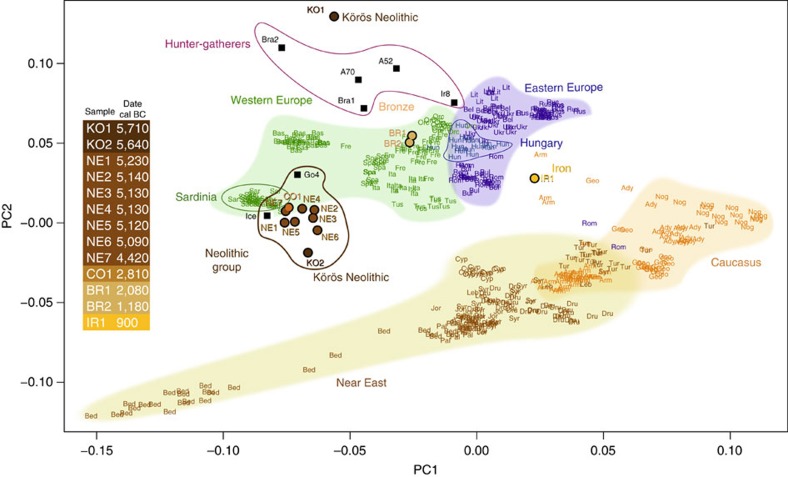
PCA of the ancient Hungarian time series. Combined plot of Principal Component Analyses performed on the ancient Hungarian time series of observed genotypes compared with European, Caucasian and Near Eastern modern populations. Published ancient genomic data are plotted as squared points: Ice^4^, Go4, A70, A52 and Ir8 (ref. 2), Bra1 and Bra2 (ref. 1). Modern population labels are: Ady, Adygei; Arm, Armenians; Bas, Basque; Bed, Bedouins; Bel, Belorussians; Bul, Bulgarians; Cyp, Cypriotes; Dru, Druze; Fre, French; Geo, Georgians; Hun, Hungarians; Ita, Italians; Jor, Jordanians; Leb, Lebanese; Lit, Lithuanians; Nog, Kuban Nogays; Orc, Orcadians; Pal, Palestinians; Rom, Romanians; Rus, Russians; Sar, Sardinians; Spa, Spaniards; Syr, Syrians; Tur, Turks; Tus, Tuscans; Ukr, Ukranians.

**Figure 3:**
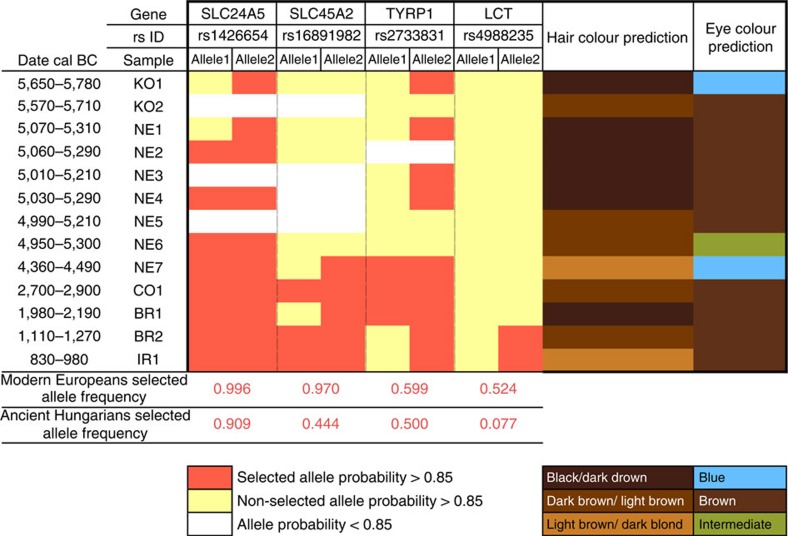
Selective sweeps. Genotypes of four SNPs associated with selective sweeps in Europe and predicted hair/eye colour for Ancient Hungarian samples. Observed data are reported for the two high-coverage samples (NE1 22.1 × and BR2 21.3 × ) while imputed genotypes are reported for all samples with lower coverage (either ~1 × or ~0.1 × ).

Based on these analyses, our samples can be divided into four sets that are located in different regions of the PCA. Our oldest sample, Körös Neolithic (KO1) (5,650–5,780 cal BC) was excavated from a short-lived agricultural settlement, perhaps spanning only two generations, at the northern range limit of the first Neolithic (Körös) cultural complex in Southeast Europe^21^. Despite its early Neolithic farming context, this genome falls towards the hunter-gatherer vicinity of the PCA plot (Fig. 2). In contrast, sample KO2, which is contemporaneous to KO1 (5,570–5,710 cal BC) and also from a Neolithic Körös Culture site only ~70 km distant, clusters with later Neolithic individuals (Fig. 2 and Supplementary Fig. 1).This marked genomic dichotomy between KO1 and KO2 suggests direct contact between indigenous hunter-gatherers and Neolithic communities as suggested previously^2^,^8^,^22^. The outlying Neolithic individual, KO1, was a blue-eyed male (Fig. 3) and his Y-chromosome lineage, I2a, matches the only haplogroup reported to date in Mesolithic Central and Northern Europeans^5^,^8^ (*n*=6).

Our Neolithic genomes all cluster with affinity to Southern Mediterranean individuals, particularly Sardinians, echoing the results of previous direct analyses of European Neolithic and post-Neolithic genomes^2^,^6^,^8^. This affinity persists through nine successive time points in our data, including a diversity of Neolithic cultures. In contrast, we observe high mtDNA diversity during this period, as previously observed in Central Europe^23^. Affinities of our observed Y-chromosome lineages (I2 and C6 haplogroups, Table 1) with a Mesolithic background^5^,^7^ and our mtDNA haplogroups with farming communities (especially the N1a haplogroup, Table 1)^24^ tentatively support the incorporation of local male hunter-gatherers into farming communities during the Central European Neolithic (Table 1), in contrast to the male-dominated diffusion of farmers suggested for the Mediterranean route^25^.

The genomic stasis of the Neolithic is subsequently interrupted during the third millennium BC coinciding with the onset of the Bronze Age. Our two Bronze Age samples, BR1 (1,980–2,190 cal BC) and BR2 (1,110–1,270 cal BC) fall among modern Central European genotypes. Within this period the trade in commodities across Europe increased and the importance of the investigated region as a node is indicated by the growth of heavily fortified settlements in the vicinities of the Carpathian valleys and passes linking North and South^26^. These two Bronze Age genomes represent the oldest genomic data sampled to date with clear Central European affinities.

A third genomic shift occurs around the turn of the first millennium BC. The single Iron Age genome, sampled from the pre-Scythian Mezőcsát Culture (Iron Age (IR1), 830–980 cal BC), shows a distinct shift towards Eastern Eurasian genotypes, specifically in the direction of several Caucasus population samples within the reference data set. This result, supported by mtDNA and Y-chromosome haplogroups (N and G2a1, respectively, both with Asian affinities) suggests genomic influences from the East. This is supported by the archaeological record which indicates increased technological and typological affinities with Steppe cultures at this time, including the importation of horse riding, carts, chariots and metallurgical techniques^26^. Modern Hungarians occupy an intermediate position between the IR1 and more Western Bronze Age genomes, most likely reflecting the continuation of admixture in the Central European gene pool since this time.

### Imputation of ancient genomes

**Figure 4:**
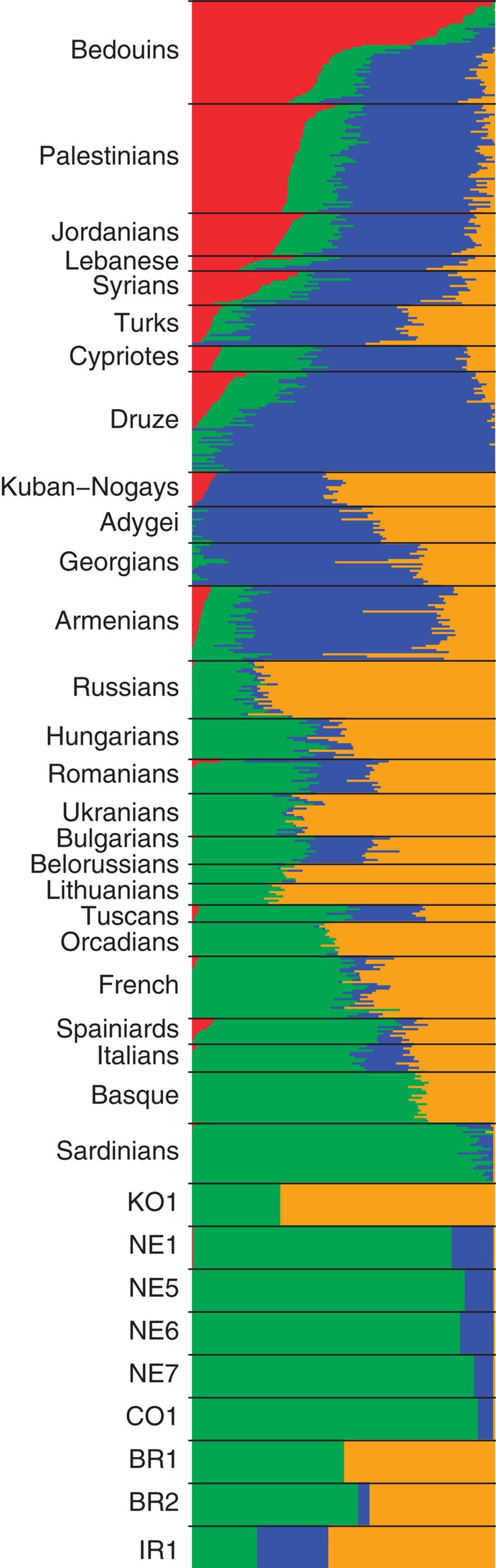
Ancient Hungarians ADMIXTURE plot. ADMIXTURE analysis (*K*=4) of the nine 1 × imputed samples along with 552 modern reference samples (HGDP+) using a LD (*r*^2^<0.2) filtered data set of 60,824 SNPs.

The information content of low-coverage genome sequences may be leveraged using imputation with a phased reference panel to achieve genome-wide diploid genotypes and enable richer data analyses^27^. To test this approach in the context of palaeogenomic data we used the 1,000 Genomes Project phased reference data to impute 5,309 and 6,159 well-characterized SNP genotypes on chromosome 22 from a range of downsampled coverage levels of NE1 and BR2, respectively, and compared calls with those made directly from their full genome data (Supplementary Methods)^27^,^28^. Considering an imputed 1 × sample of NE1 and imposing a genotype probability threshold of 0.99, 78% of these loci remained, with diminishing return from increasing coverage in the subsample (Supplementary Fig. 5). Of the imputed genotypes, 99.20% (99.18% of heterozygotes) matched the observed high-coverage calls, validating this approach for expanding our data (Supplementary Figs 5–9). With the more recent BR2 genome, imputation from 1 × coverage allowed 80.0% of loci to be called at a 0.99 thereshold with 99.33% (99.05% of heterozygotes) match to high-coverage calls. Therefore, we imputed genome-wide genotypes for each of the low-coverage genomes and, after intersection with modern SNP data, called a total of 151,407 high-quality diploid loci across all samples. These data were used in an ADMIXTURE analysis^29^, which reaffirmed the clustering and temporal shifts in affinity observed in the PCA visualization (Fig. 4).

**Figure 5:**
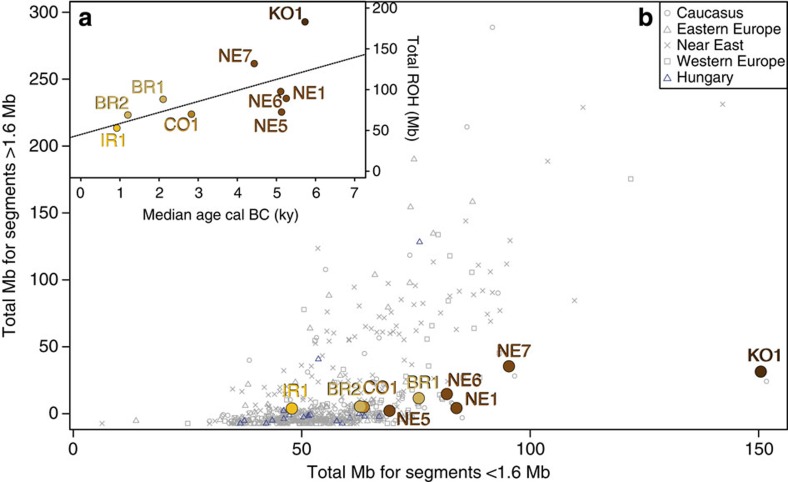
Short versus long ROH. (**a**) Regression between age (median value, cal. BC) and total Mb of run of homozygosity (*r*^2^=0.4042, *P*-value=0.06573). (**b**) Short (<1.6 Mb) versus long (>1.6 Mb) runs of homozygosity based on 151,407 autosomal SNPs for the 9 ancient Hungarian samples compared with European, Near Eastern and Caucasian populations (Supplementary Methods).

These data also allowed us to estimate the fraction of each genome under runs of homozygosity^30^ (Fig. 5), which gives information about past demography. Long contiguous homozygous segments within a genome are indicative of recent endogamy while shorter runs result from older manifestations of small ancestral population size^31^. Unlike a small subset of modern genomes, no ancient genome in our analysis showed a clear excess of long runs of homozygosity (ROH) (>1.6 Mb), suggesting each to have been outbred. However, a clear temporal trend was evident in the extent of ROH, especially shorter ROH, with the Bronze Age and IR1 individuals falling within the bulk of modern values, Neolithic specimens tending towards the upper end of this range and the Early Neolithic Körös specimen, KO1 forming a clear outlier. This suggests an unusually restricted ancestral population size for KO1 (we note that low heterozygosity was also found within a 8,000 year old hunter-gatherer from Luxembourg^5^), supporting the inference that he represents an exogenous individual in a farming settlement. A possible criticism of this approach is that bias against heterozygous calls and the existence of ancient haplotypes that are absent from the reference genomes may impede analyses built on imputed data; our high-coverage ancient Neolithic genome, NE1, falls outside PCA clusters of the 1,000 Genomes reference individuals, along with our KO1 and IR1 samples (Supplementary Fig. 8). Therefore, we further analyzed genome-wide imputations of 0.5 × , 1 × and 2.5 × coverage samples of the NE1 and BR2 genomes. In both PCA and ROH plots of genotypes from these, the positions from each replicate were highly similar to those generated from high-coverage SNP calls (Supplementary Figs 6 and 7).

### Genotypes under selection

Imputation permitted us to follow the temporal dynamics of genetic variants that are believed to have been under selection. Of two skin pigmentation loci known to have swept to fixation during European prehistory^32^,^33^, the light pigmentary variant of *SLC24A5* is present from the earliest of our samples and is homozygous from the Middle Neolithic onwards, whereas the light pigmentary variant of *SLC45A2* only appears towards the later half of our transect with the first homozygote genotype in the Copper Age (Fig. 3). Both *SLC24A5* and *SLC45A2* exhibited an ancestral homozygous state in Mesolithic specimens of Central^5^ and Western Europe^7^, while *SLC24A5* had the derived state in a Central European Neolithic individual^5^. Our temporal transect suggests separate selective sweeps at these two pigmentary loci, acting over a millennium apart. The selected variant at a third pigmentary locus with a proposed adaptive history in Europe, *TYRP1*, also shows some tendency to higher prevalence in later samples. This temporal transition towards lighter pigmentation is also seen with hair where colours and shades estimated from SNPs used in the forensic Hirisplex system grade from black/dark brown in earlier samples to light brown and dark blonde in later individuals (Fig. 3).

One of the strongest signals of selection within human genome variation is that around the lactase persistence allele in Europeans; a response to a dietary focus on raw milk from domestic cattle. It has been postulated that this allele first underwent selection 5,500 years BC, possibly in association with the Neolithic LBK culture within Central Europe^34^. Here in our temporal sequence, its appearance is delayed until the more recent of our Bronze Age individuals, who lived only ~1,000 years BC.

## Discussion

The extension of population genomics into the temporal dimension is an exciting recent development in the field of human evolution but the low endogenous DNA content of most archaeological bones is a major constraint, even with falling sequencing costs, accessing whole genomes from samples comprising <1% target genome is often prohibitive. We have shown that for a range of samples from different sites and time depths up to ~8,000 years, excellent yields of >50% are typical from extractions of the petrous portion of the skull temporal bone. Where tested, this contrasts significantly with yields from other skeletal parts from the same individual, despite similar taphonomic conditions. We suggest that the high density^17^ of the petrous bone results in reduced bacterial and chemical-mediated post-mortem DNA decay. We also show that, at least for Europeans, imputation of 1 × genome coverage sequences can give genome-wide diploid calls for ~80% of genome-wide SNPs, at ~99% accuracy, greatly leveraging their information content. These data can be used to examine SNPs of particular phenotypic interest and make whole genome analyses such as examination of ROH, ADMIXTURE and PCA analysis possible. It is important to note, though, that other methods may be sensitive to biases among SNPs as to which are imputable and that samples of higher divergence from the reference populations may impute with lesser accuracy.

Genome-wide imputation offered the opportunity to assess phenotypic change through time from low-coverage genomes. Our samples show a tendency towards lighter pigmentation through and after the Neolithic. In particular we examined three pigmentation SNPs that display European-specific selective sweeps that are presumed to facilitate vitamin D synthesis and estimated as having occurred within the last 11,000–19,000 years^33^. We surmise that these sweeps occurred more recently, within the time depth of our transect, with *SLC24A5* showing the earliest fixation (~5,000 BC), while *SLC45A2* and *TYRP1* were not found in homozygous individuals until the Late Neolithic (~4,000–3,000 BC). Wilde *et al.*^32^ also found intermediate frequencies for *SLC45A2* in ancient Ukranian Eneolithic and Early Bronze Age samples. The strongest dietary adaptive signal in the human genome is the highly structured global distribution and extended homozygosity around the lactase persistence allele in European genomes^35^. Selection on this variant was undoubtedly driven by dairying, but despite evidence for milk residues in ceramic vessels from a Körös context in the 6th millenium BC (ref. 36) this variant remains absent throughout the 10 Neolithic/Copper Age stages of our transect. Absence of the lactase persistence allele has been reported before from Neolithic specimens^37^,^38^, although the selective sweep has been modelled as originating between Central Europe and the Balkans ~4–6,000 years BC (ref. 34). Its absence here until the late Bronze Age, ~1,000 years BC, suggests a more recent dating of this extremely interesting episode in the dynamic history of European genomes.

Beyond inferences about individual phenotypes, we have used our results to examine the population genetic affinities of a temporal transect of genome sequences from burials on the Great Hungarian Plain, a region of high archaeological significance for major European cultural transitions. We investigated samples across a diversity of archaeological cultures and show evidence for major shifts in genome affinity accompanying the advents of the Neolithic, Bronze and Iron Ages, strongly implying that these changes in material culture were accompanied by substantial migrations. The Neolithic genomes reported here accord with prior German, Scandinavian and Alpine early farmer genomes in showing an immigrant signature of Southern Mediterranean affinity^2^,^5^,^6^,^8^. However, an intriguing finding is that of a single individual with a strongly Mesolithic genomic signature within the context of the Körös culture, part of the earliest Neolithic of Southern Europe. This is the earliest genetic indication of contact between these two subsistence strategies. In the Middle and Late Hungarian Neolithic local Mesolithic influence is further discernible through the appearance of mtDNA and Y-chromosome haplogroups typical of European hunter-gatherer populations, concurring with other evidence for admixture in the ancestry of European farmers^5^,^8^,^22^,^23^.

Similar to the Tyrolean Copper Age iceman^6^ our Copper Age (Baden Culture) sample shows similarity to Neolithic genomes, in accordance with archaeological continuity in the region. In contrast, the Bronze Age genomes shift towards an affinity to Central Europe, suggesting migratory influence from the North. The single pre-Scythian IR1 genome shows another shift towards migration from the East. Altogether, our results accord with archaeological perspectives that link these major transitions in European material culture to population movements rather than cultural diffusion alone.

## Methods

### Samples

We analyzed 23 samples belonging to 13 ancient individuals from Eastern Hungary (Table 1; Supplementary Note 1). All individuals have been directly dated, spanning from the Early Neolithic (~5,700 cal BC) to the IR1 (~800 cal BC) (Supplementary Fig. 1; Supplementary Table 1; Supplementary Note 1). For 7 of the 13 individuals investigated (Supplementary Fig. 2; Supplementary Table 2; Supplementary Methods) we compared dental crowns (IDs 8.2, 14.4, 14.7) and roots (IDs 8.3, 14.5, 14.8), ribs (IDs 8.5, 10.2), metacarpal (ID 10.6) and metatarsal portions (ID 10.4) to petrous bone parts within the same individual (Fig. 1; Supplementary Table 2) and for individual NE6 we investigated two different areas of the same temporal bone (IDs 14.2, 14.3).

### Laboratory procedures

DNA extraction was carried out with ~300 mg of bone powder (Supplementary Table 3) and following a silica-column-based protocol based on the study by Yang *et al.*^39^, as modified by MacHugh *et al.*^40^ (Supplementary Methods).

Libraries were constructed following the study by Meyer *et al.*^41^ with few modifications (Supplementary Methods). Indexing PCRs were performed using Accuprime Pfx Supermix (Life Technology), purified using QIAGEN silica columns and the quality was assessed on an Agilent 2100 Bioanalyzer. All libraries were first screened on an Illumina MiSeq platform (50 bp single-end) and libraries from petrous bones were further sequenced on an Illumina HiSeq 2000 (100 bp single-end sequencing) (Supplementary Tables 2 and 3).

### Genome mapping and SNP calling

Raw reads were filtered based on the indices used and the adapter sequences were trimmed using cutadapt v1.3 (ref. 42) (Supplementary Methods). Two bases of the reads at the 5′ and 3′ ends were trimmed before mapping, following the study by Meyer *et al.*^43^, using seqtk ( https://github.com/lh3/seqtk), for a final minimum length of 30 bp.

Sequence reads were aligned independently to the nuclear DNA (GRCh37) and mtDNA (rCRS, NC_012920.1) using Burrows–Wheeler Aligner^44^ disabling the seed option. Duplicate reads were removed using Samtools^45^ and indels were realigned using Genome Analysis Toolkit (GATK) RealignerTargetCreator and IndelRealigner^46^. Genomic depth of coverage was calculated using depth-cover ( https://github.com/jalvz/depth-cover).

BAM files obtained from different sequencing lanes were merged using the MergeSamFiles Picard tool ( http://picard.sourceforge.net). Duplicates were further removed and filtered by using mapping quality >30 (Supplementary Methods; Supplementary Table 3; for mtDNA see Supplementary Methods).

To explore the ancient data in the context of modern variation we called genotypes at all positions that overlapped with the HGDP+ data set (Supplementary Methods), which includes European, Caucasian and Near Eastern populations^18^,^19^,^20^. Different SNP calling procedures were followed for the high-coverage and low-coverage data (Supplementary Methods).

### Authenticity of results

All stages of the genetic analysis, up to the library amplification set-up, were carried out in dedicated ancient DNA facilities at Trinity College Dublin, Ireland. Standard precautions to avoid contamination were taken, including wearing coveralls, mask, hair cover, shoe covers and double gloves. Working surfaces and all materials were frequently cleaned with DNA-ExitusPlus and subsequently ultraviolet-irradiated.

The high percentages of human DNA point to very good preservation in all samples. Nevertheless, contamination was further controlled and estimated by: (a) independent replication, (b) sequencing of negative controls, (c) estimation of molecular damage and sequence length, (d) mtDNA contamination estimates and (e) X chromosome contamination estimates in males (Supplementary Methods).

### Sex determination and uniparental ancestry

Sex was determined by analyzing the ratio of X- to Y-chromosome reads following the study by Skoglund *et al.*^47^ (Supplementary Methods, Supplementary Fig. 4). Samples were mapped to the mtDNA genome and filtered as above. BAM files were analyzed with the online tool MitoBamAnnotator^48^ (Supplementary Methods). Haplogroups were obtained with Haplogrep^49^ based on the build 15 phylogeny of PhyloTree^50^ (Supplementary Table 12).

Y-chromosome haplogroups were determined for all male samples by analyzing the filtered SNP set of the International Society of Genetic Genealogy Y-DNA Haplogroup Tree 2014 (ISOGG, Version 9.22, Supplementary Methods; Supplementary Table 13).

### Imputation

We evaluated the potential of whole genome imputation to infer diploid genotypes from low-coverage genomes by randomly subsampling our high-coverage genomes (NE1 and BR2) to a series of coverages ranging from 0.1 to 5 × using SAMtools^45^ (Supplementary Methods; Supplementary Fig. 5). Using GATK’s UnifiedGenotyper tool, genotype likelihoods were called from subsampled sequence data at 28,627,866 autosomal SNP sites with a minor allele count >1 in the phase 1 integrated release of the 1,000 Genomes Project^28^. Genotype likelihoods were called only for the alleles observed in the 1,000 Genomes Project data and were converted from log space to linear space and parsed to Beagle format, assigning equal likelihoods (0.3333) to sites with no spanning sequence data. Genotype likelihoods were also reset to 0.3333 for any genotype that could have derived from a deaminated cytosine residue.

We used data from these studies to inform our choice of target depth for our low-coverage genomes, then applied the same imputation methodologies to these real data sets (11 individuals; coverage 0.1–1 × ) to infer accurate diploid genotype calls. For genome-wide analyses (PCA and ADMIXTURE), genotype probabilities were converted to PLINK-format BED data, imposing a genotype probability threshold of 0.99. For single-locus analysis of selective sweeps, a genotype probability threshold of 0.85 was used (Supplementary Methods).

### PCA and ADMIXTURE

We performed individual PCA in the context of the HGDP+ (Supplemetaty Methods) data set on all observed genotyping of the 13 samples using SMARTPCA (refs 2, 51) (Supplementary Methods). We used a Procrustes approach as in the study by Skoglund *et al.*^2^ to transform the PCA coordinates of each sample and to plot them together in the context of the HGDP+ data set (Fig. 2; Supplementary Tables 4 and 5). PCA was also performed on 1 × imputed data with a probability cutoff of 99% (151,407 shared SNPs, Supplementary Fig. 9; Supplementary Methods).

ADMIXTURE (ref. 29) and NgsAdmix (ref. 52) were used to estimate the ancestral genetic components of nine ancient samples (seven ~1 × and two 1 × downsampled ~20 × genomes) together with 552 modern samples after filtering the SNP data set on imputed and observed genotypes, respectively (Supplementary Methods).

### Runs of homozygosity

ROH analysis was performed with nine ancient imputed genomes, two of them imputed from high-coverage genomes (samples NE1 and BR2) subsampled to ~1 × and seven originally ~1 × genomes (samples KO1, NE5, NE6, NE7, CO1, BR1 and IR1). Imputed genotypes were called if their genotype probability was ≥0.99. PLINK (ref. 53) was used to merge these imputed genotypes with the HGDP+ data set (Supplementary Methods), giving a total of 151,407 SNPs shared between all samples. We also estimated the correlation between sample age and the total length of ROH (Fig. 5a; Supplementary Methods).

### Selective sweeps and phenotypes

We examined SNPs in four genes implicated in pigmentation and which have been suggested to have undergone selective sweeps in European prehistory (Fig. 3). Imputed genotypes from each low-coverage sequenced ancient individual were considered along with directly observed alleles for the two high-coverage sequences.

Genotypes were imputed for three pigmentation genes (SLC24A5, SLC45A2, associated with skin colour, and TYRP1, associated with iris and hair pigmentation) in which three SNPs (rs1426654, rs16891982 and rs2733831, respectively) have been specifically selected in Europeans, and the lactase persistence gene in Europeans associated with the T allele at SNP rs4988235 (Supplementary Methods).

We also implemented two models for phenotype prediction developed in forensic science for our pool of ancient genomes (Supplementary Methods): the 8-plex^54^,^55^ and the Hirisplex^56^ systems. We used the former to infer the pigmentation of skin and the latter for eye and hair colour prediction.

## Author contributions

R.P., M.H. and D.G.B. supervised the study. C.G., E.R.J., M.D.T., R.L.M., G.G.-F. and D.G.B. analyzed genetic data. R.P., L.D., I.K., I.P., A.A., J.D., P.R. provided archaeological samples and input about the archaeological context, R.P., T.F.G.H., A.W. and L.D. provided and analyzed radiocarbon determinations, C.G., E.R.J. and G.G.-F. processed ancient DNA and prepared sequencing libraries. V.M. conducted sequencing. C.G., R.P. and D.G.B. wrote the manuscript with contributions from all co-authors.

## Additional information

**Accession codes:** Raw alignment data have been deposited in GenBank/EMBL/DDBJ Sequence Read Archive (SRA) under the accession code SRP039766.

**How to cite this article:** Gamba, C. *et al.* Genome flux and stasis in a five millennium transect of European prehistory. *Nat. Commun.* 5:5257 doi: 10.1038/ncomms6257 (2014).

## Supplementary Material

Supplementary Information Supplementary Figures 1-10, Supplementary Tables 1-17, Supplementary Notes, Supplementary Methods and Supplementary References

## Figures and Tables

**Table 1:** Result summary from 13 Hungarian petrous bone samples.

**Individual**	**Mean coverage**	**Human DNA (%)**	**Period and culture**	**Site**	**Age (cal BC)**	**Sex**	**mtDNA haplogroup**	**Y Haplogroup**
KO1	1.24	62.80	E. Neol. Körös	Tiszaszőlős-Domaháza	5,650–5,780	M	R3	I2a
KO2	0.13	10.13	E. Neol. Körös	Berettyóújfalu-Morotva-liget	5,570–5,710	F	K1	—
NE1	22.12	86.85	M. Neol. ALP	Polgár-Ferenci-hát	5,070–5,310	F	U5b2c	—
NE2	0.19	45.85	M. Neol. ALP Esztár Group	Debrecen Tócópart Erdõalja	5,060–5,290	F	H	—
NE3	0.13	37.60	M. Neol. Bükk Culture	Garadna	5,010–5,210	F	X2b	—
NE4	0.10	15.16	M. Neol. Tiszadob-Bükk Culture	Polgár-Ferenci-hát	5,050–5,2905,030–5,280	F	J1c	—
NE5	1.04	71.02	M. Neol. Late ALP	Kompolt-Kigyósér	4,990–5,210	M	J1c1	C6
NE6	1.18	80.36	M. Neol. LBK Culture	Apc-Berekalja I.	4,950–5,300	M	K1a3a3	C6
NE7	1.14	62.81	L. Neol. Lengyel Culture	Apc-Berekalja I.	4,360–4,490	M	N1a1a1a	I2a
CO1	1.13	34.57	L. Copper Age, Baden Culture	Apc-Berekalja I.	2,700–2,900	F	H	—
BR1	0.81	70.85	E. Bronze, Makó Culture	Kompolt-Kigyósér	1,980–2,190	F	K1c1	—
BR2	21.25	55.31	L. Bronze, Kyjatice Culture	Ludas-Varjú-dűlő	1,110–1,270	M	K1a1a	J2a1
IR1	1.31	56.37	Iron Age, Pre-Scythian Mezőcsát Culture	Ludas-Varjú-dűlő	830–980	M	G2a1	N

ALP, Alföld Linear Pottery; E.,early; F, female; KO1, Körös Neolithic; L., late; LBK, Linearbandkeramik; M, male; M., middle; mtDNA, mitochondrial DNA; Neol., Neolithic.

Dates are in calibrated years BC at 2 s.d., 95.4% confidence interval calibrated using OxCal 4.2 and rounded to the decade. For the individual NE4 two dates were obtained.
