# Supplementary material for: Genome flux and stasis in a five millennium transect of European prehistory
Source: Nat Commun. 2014 Oct 21;5:5257. doi: 10.1038/ncomms6257 (PMC4218962; doi:10.1038/ncomms6257)
Supplement: Supplementary Information — Supplementary Figures 1-10, Supplementary Tables 1-17, Supplementary Notes, Supplementary Methods and Supplementary References [file ncomms6257-s1.pdf]

SUPPLEMENTARY FIGURES

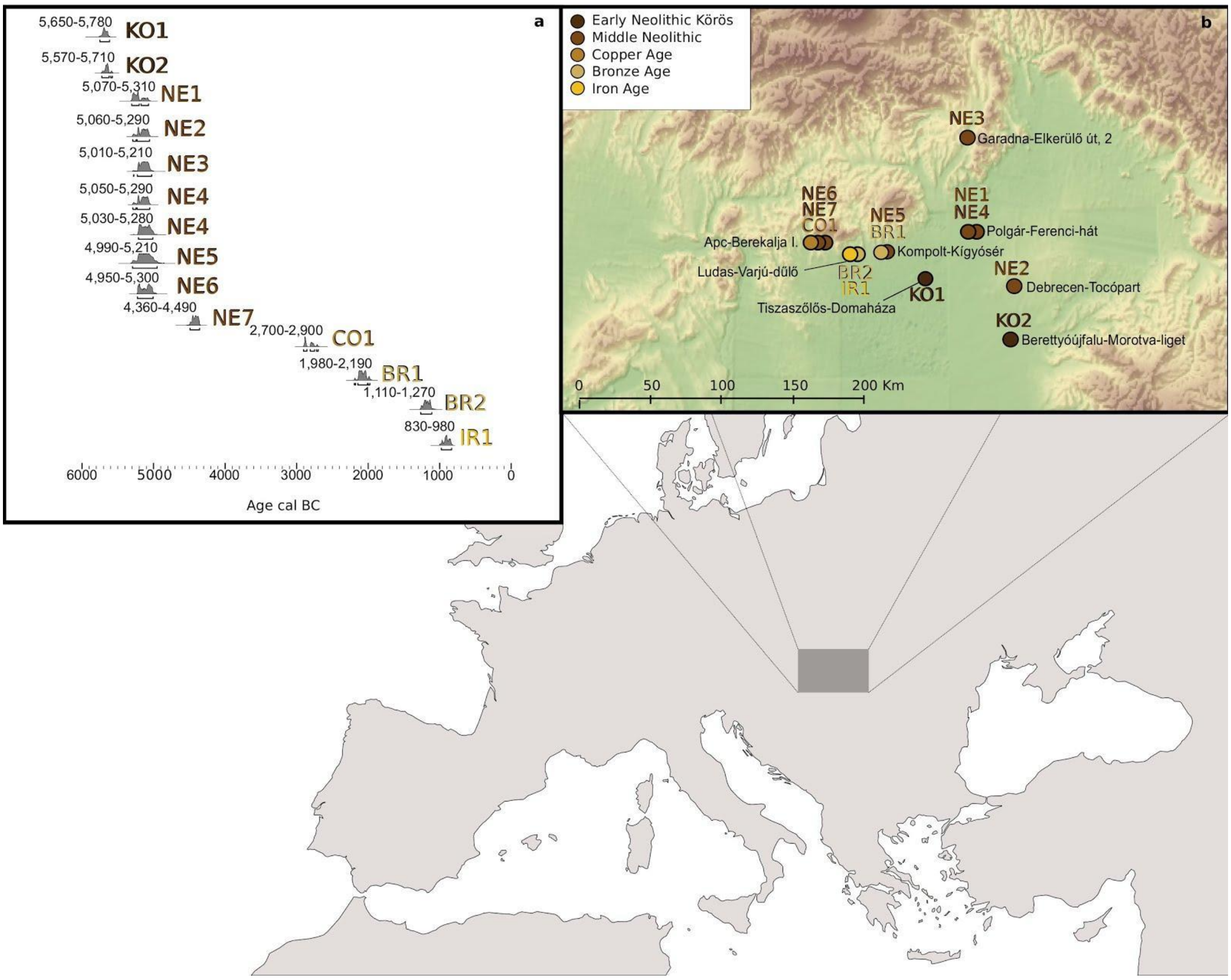

Supplementary Figure 1. **a**) Radiocarbon dates curves and **b**) geographic location of the samples studied.

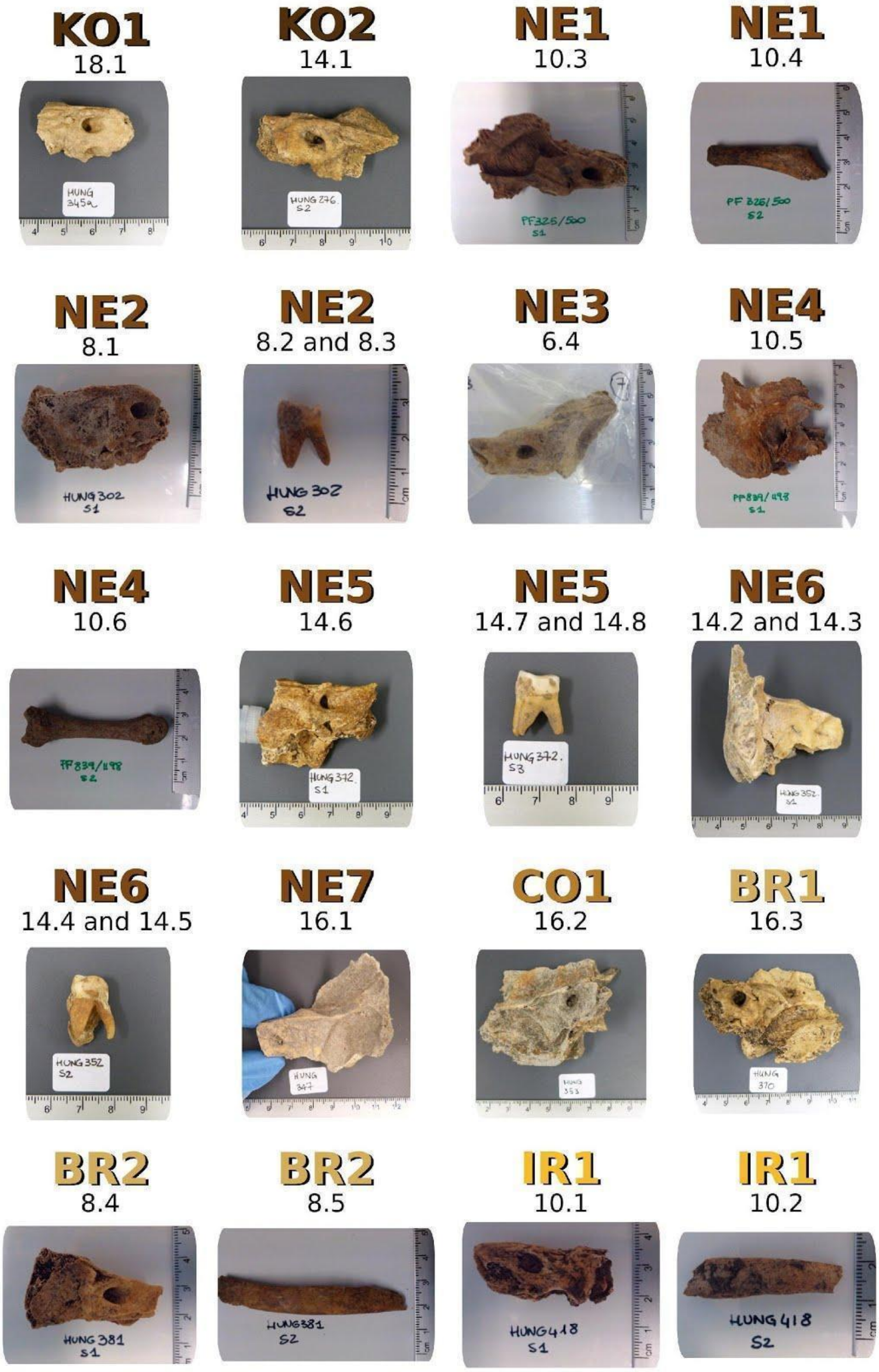

Supplementary Figure 2. Pictures of samples analyzed. The name of each individual is displayed in colour while the corresponding number refers to the specific sample (IDs). Duplication of sample names indicates that two different skeletal elements were analyzed from the same individual.

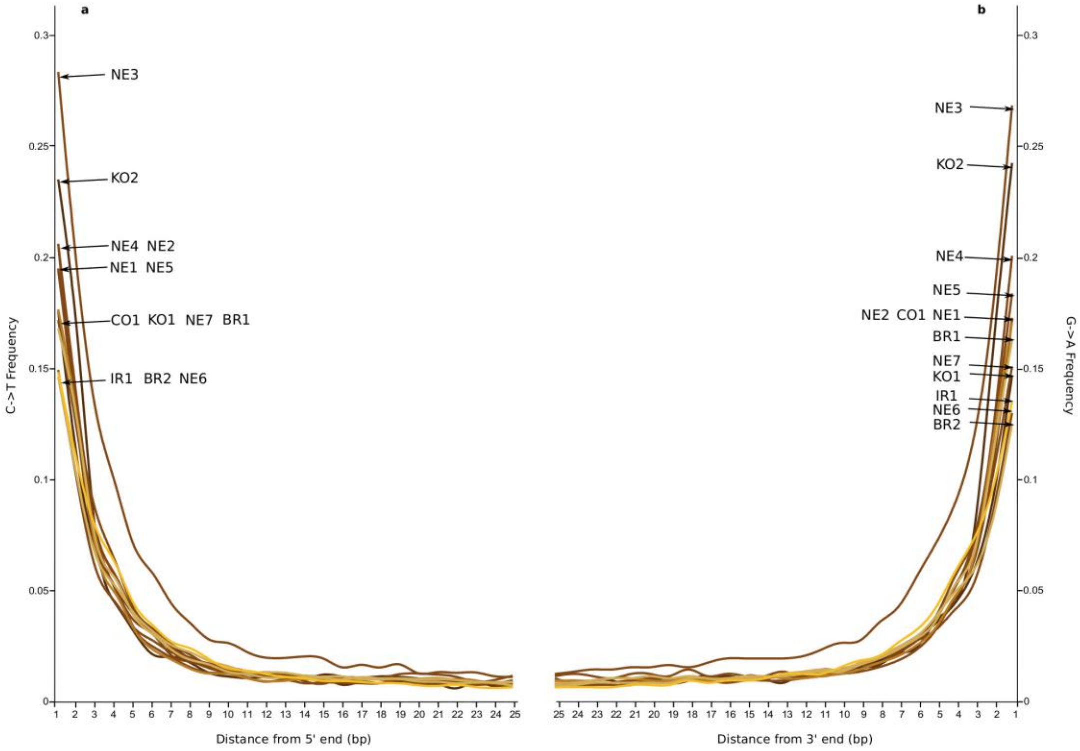

Supplementary Figure 3. **a**) Frequencies of C to T and **b**) G to A transitions per sample at positions 1 to 25, from the 5' and 3' end respectively, calculated from a subsample of 500,000 reads per sample with mapDamage 2.0 [41,88]..

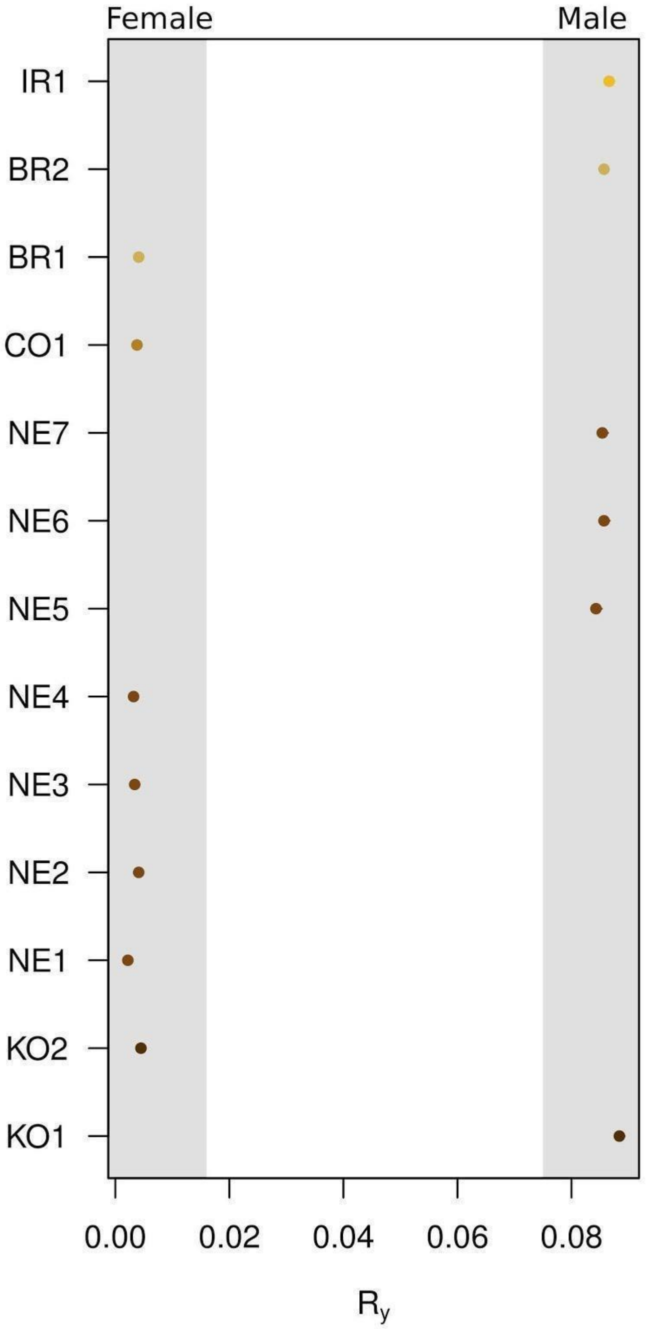

Supplementary Figure 4. Ratio of the fraction of Y chromosome reads to the fraction of reads aligning to both sex chromosomes (R_y) in all studied samples. Error bars (95% confidence interval) are included but not visible due to low standard error. The border of the grey regions illustrates upper and lower bounds for sex assignment [42]..

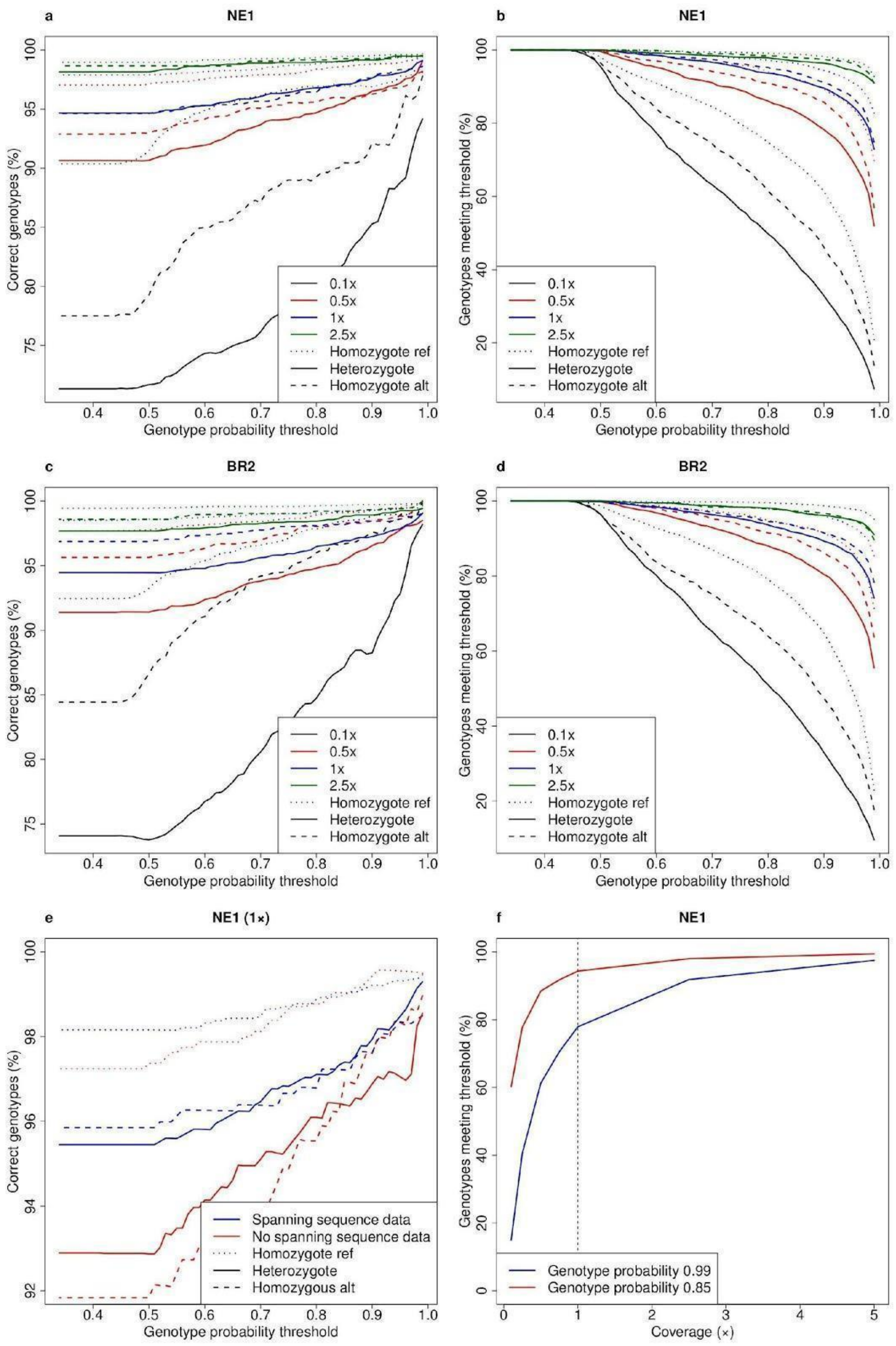

Supplementary Figure 5. Accuracy of imputation. **a**) and **c)**: Genotypes imputed from subsampled data were compared against high-coverage genotypes for samples NE1 and BR2 and called "Correct" if they matched. Four representative subsampled coverages (0.1× - 2.5×) are presented using different line colors. These are broken down by genotype call, delineated by line texture, and at each level heterozygote calls show least accuracy. **b**) and **d**): Proportions of imputed genotypes retained at each coverage with imposition of a range of genotype probability thresholds. **e)** Proportion of correct (compared with high-coverage data) genotype calls following imputation in NE1 1× data as a function of genotype probability threshold, separated according to whether sequence data spanned the SNP site. **f**) Percentage of genotypes meeting two representative imputed genotype probability thresholds for all the analyzed subsampled coverages. We chose 1× as a suitable target coverage for our imputed genomes as it provided adequate data following imputation without extra investment in sequencing.

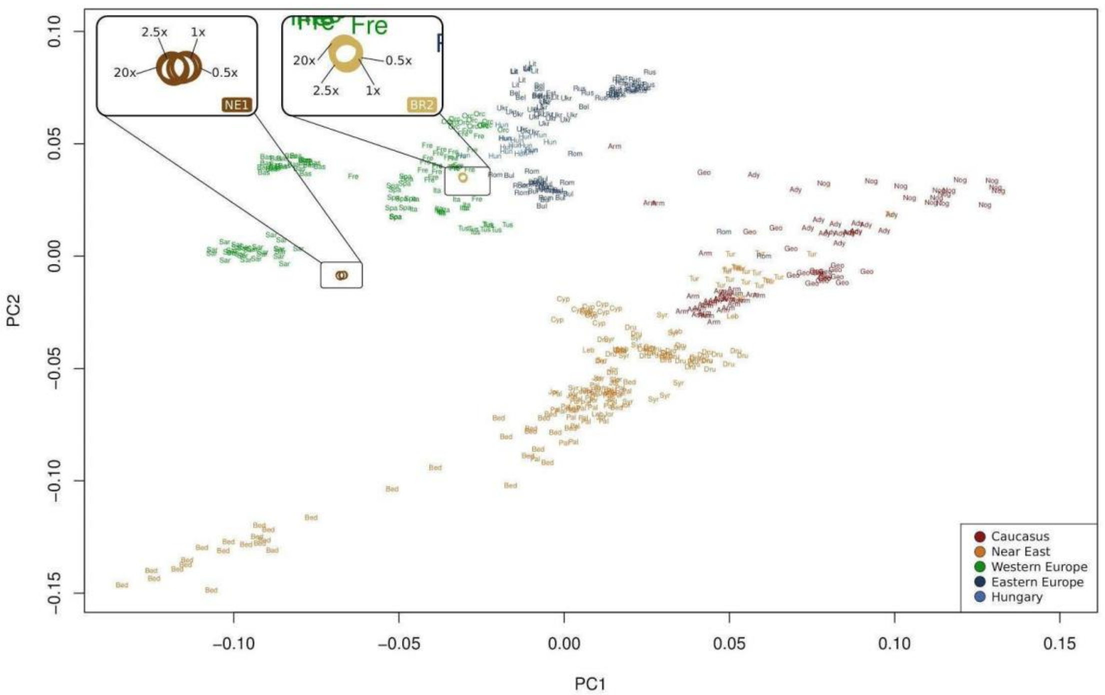

Supplementary Figure 6. Plot of principal components analyses performed on HGDP+ individuals combined with NE1 or BR2, using high-coverage genotype calls (~20×) or genotypes imputed from subsampled data (0.5×, 1× and 2.5×). Modern population labels are described in Fig. 1. Within the variation observed in principal components 1 and 2, high-coverage genotypes yield very similar results to genotypes imputed from subsampled data.

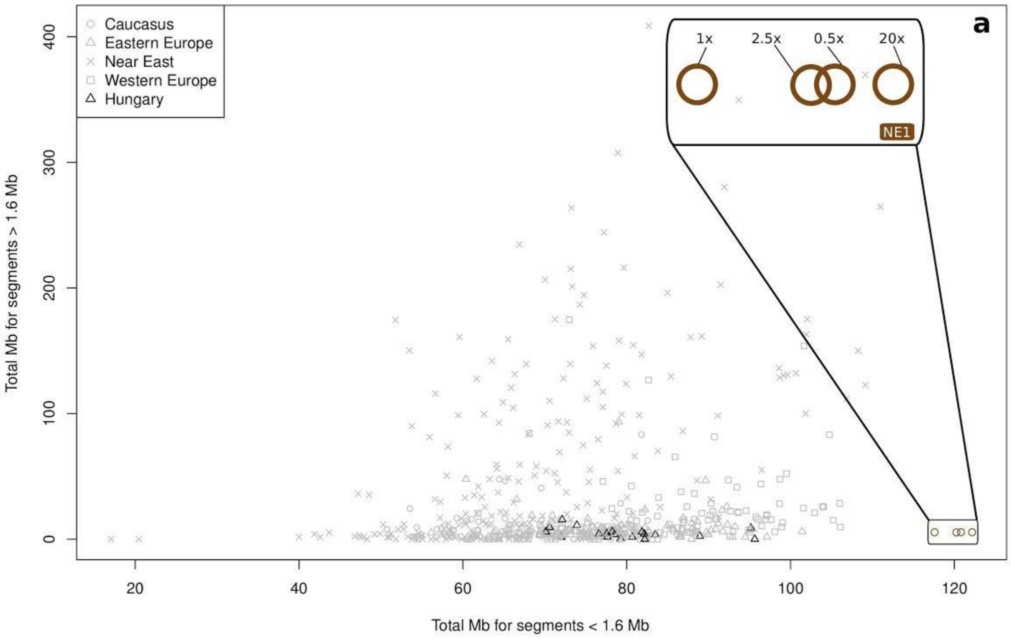

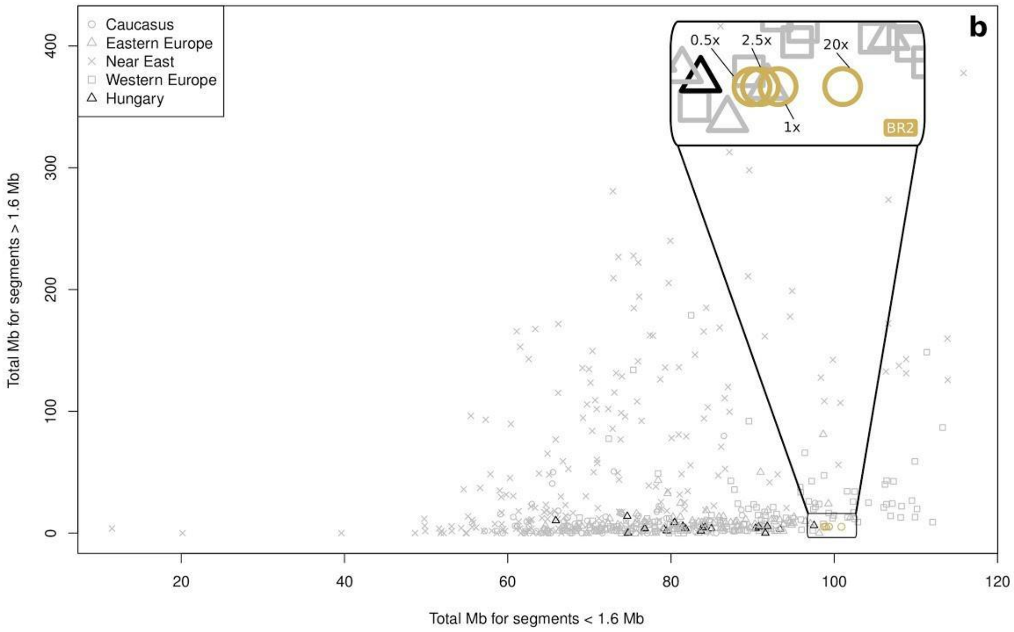

Supplementary Figure 7. Short (below 1.6Mb) versus long (above 1.6 Mb) runs of homozygosity performed on HGDP+ individuals combined with NE1 or BR2, using high-coverage genotype calls (~20×) or genotypes imputed from subsampled data (0.5×, 1× and 2.5×) and based on 226,915 (NE1) and 241,883 (BR2) autosomal SNPs.

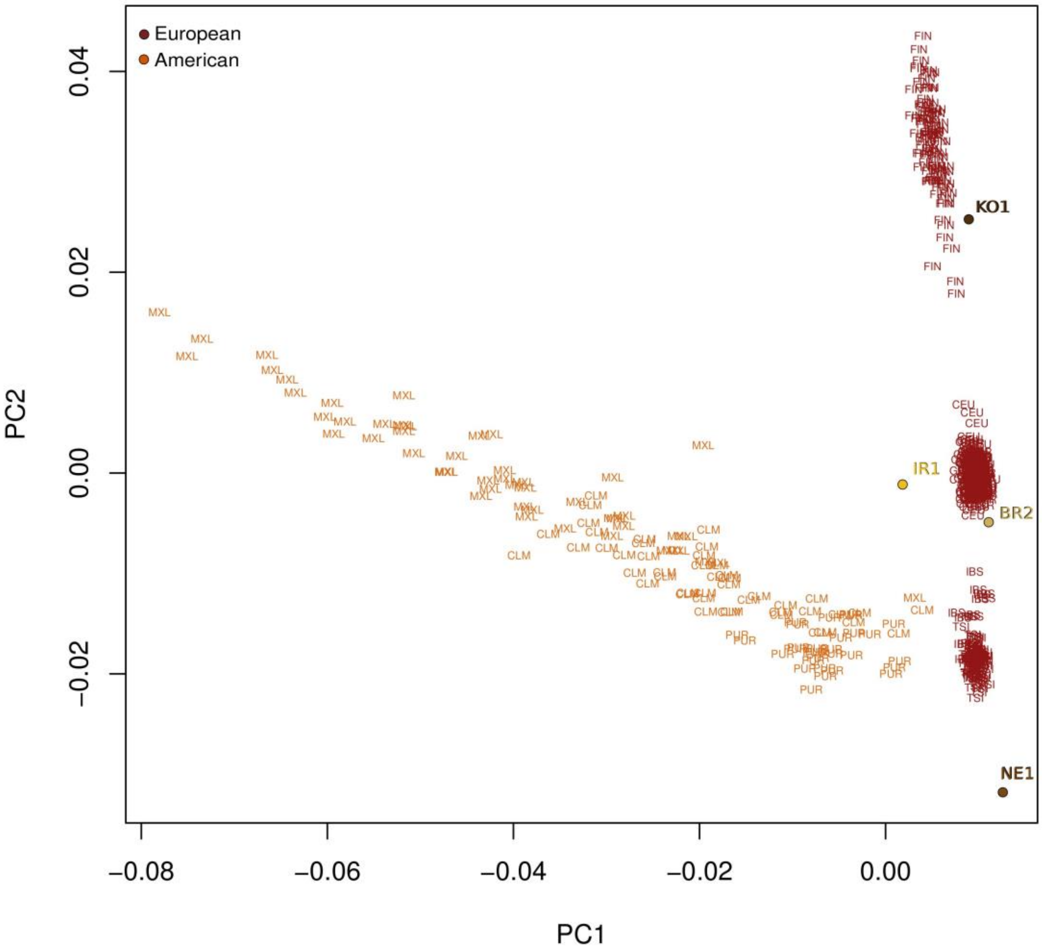

Supplementary Figure 8. Plot of principal components analyses performed on European and American populations from the 1000 Genomes database [28] combined with the outlier 1× coverage samples (KO1, IR1) in the observed PCA (**Fig.1**) and the high coverage samples NE1 and BR2 subsampled to 1×.

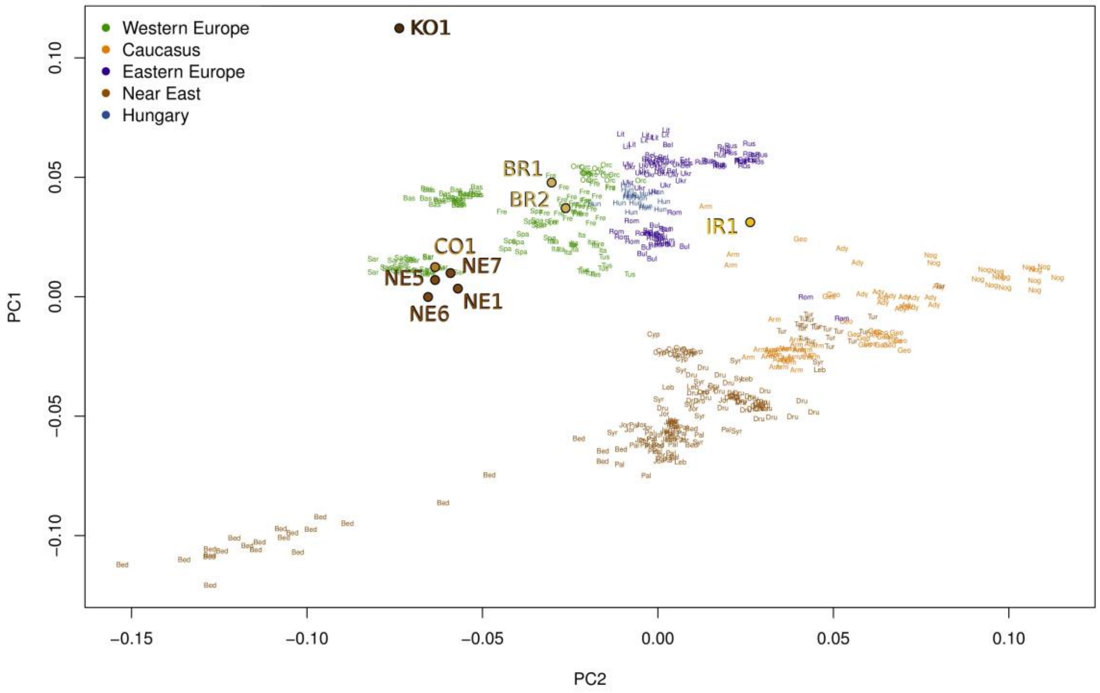

Supplementary Figure 9. Plot of principal components analyses performed on HGDP+ individuals combined with imputed low coverage ~1× samples (KO1, NE5, NE6, NE7, CO1, BR1, IR1) and imputed subsampled data (also 1×) from the two high coverage genomes (NE1 and BR2). Modern population labels are described in **Fig. 1**.

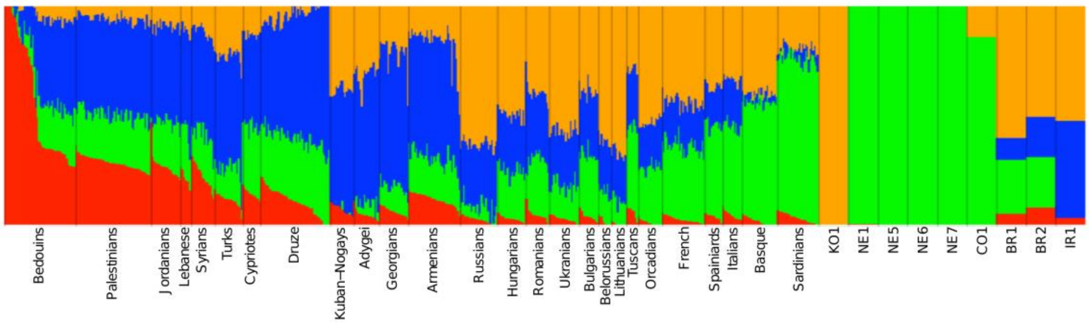

Supplementary Figure 10. Ancient Hungarians admixture plot based on observed genotypes obtained with the NgsAdmix software (K=4) of the nine ~1× samples used for imputation, along with 552 modern reference samples (HGDP+).

SUPPLEMENTARY TABLES

Supplementary Table 1. Details of sites, grave numbers and radiocarbon dates for all samples studied. All dates are in calibrated years BC at 2 SD, 95.4% confidence interval calibrated using OxCal 4.2. Two dates were obtained for NE4

Individual	Code	Site	Culture	Grave	$d^{13}C$ ‰	Date cal. BC		
						Min.	Median	Max
**KO1**	OxA-23757	Tiszaszőlős-Domaháza	E. Neol. Körös	4	-22.24	5646	5715	5781
**KO2**	OxA-28101	Berettyóújfalu-Mortva-liget	E. Neol. Körös	44 V	-20.0	5566	5643	5713
**NE1**	OxA-27861	Polgár Ferenci hát	M. Neol. ALP	325 V	-20.0	5071	5229	5306
**NE2**	OxA-27858	Debrecen Tócópart Erdőalja	M. Neol. ALP Esztár Group	1412–1761V	-19.5	5056	5144	5291
**NE3**	OxA-27732	Garadna	M. Neol. Bükk Culture	44 V	-19.7	5026	5132	5281
**NE4**	OxA-28020	Polgár Ferenci hát	M. Neol. Tiszadob-Bükk Culture	PF839/1198 V	-19.9	5011	5116	5211
**NE4**	OxA-27862	Polgar Ferenci hat	M. Neol. Tiszadob-Bukk Culture	PF839/1198 V	-20.2	5054	5141	5289
**NE5**	OxA-23763	Kompolt-Kígyósér	M. Neol. Late ALP	17/7	-19.22	4950	5116	5295
**NE6**	MAMS-14821	Apc-Berekalja I	M. Neol. LBK Culture	213/6	N/A	4992	5086	5211
**NE7**	MAMS-14819	Apc-Berekalja I	L. Neol. Lengyel Culture	2	N/A	4357	4416	4491
**CO1**	MAMS-14825	Apc-Berekalja I	L. Copper Age, Baden Culture	1231/15	N/A	2699	2806	2903
**BR1**	OxA-23799	Kompolt-Kígyósér	E. Bronze Makó Culture	7/3	-19.36	1977	2082	2194
**BR2**	OxA-27859	Ludas-Varjú-dűlő	L. Bronze Kyjatice Culture	2161.2 V	-16.0	1111	1181	1266
**IR1**	OxA-27860	Ludas-Varjú-dűlő	Iron Age, Pre-Scythian Mezőcsát Culture	2638 V	-14.4	834	904	978

E., Early; M., Middle; L., Late; Neol., Neolithic

Supplementary Table 2. Alignment statistics for all ancient samples sequenced on an Ilumina Miseq platform. Fifty base pair (bp) single-end sequencing was used. Adapters were trimmed using cutadapt as in **Supplementary Method 3.2**, retaining sequences greater than 16bp in length. Sequences were aligned to the GRCh37 build of the human genome with the mitochondrial sequence replaced by the revised Cambridge reference sequence and duplicates were removed as outlined in **Supplementary Method 3.2**. N/A indicates that the library did not pass quality assessment (**Supplementary Method 1.4**) for sequencing.

Individual	ID	Skeletal Element	Total Reads	Aligned Reads (no duplicates)	Human DNA (%)
NE1	10.4	Metatarsal bone	1,423,755	9,282	0.65
NE1	10.3	Petrous bone	1,261,313	729,556	57.84
NE2	8.3	Molar roots	1,156,574	40,696	3.52
NE2	8.2	Molar crown	1,596,570	49,026	3.07
NE2	8.1	Petrous bone	1,479,695	552,896	37.37
NE4	10.6	Metacarpal	N/A	N/A	N/A
NE4	10.5	Petrous bone	1,016,629	123,991	12.20
NE5	14.8	Molar roots	1,724,567	313,187	18.16
NE5	14.7	Molar crown	2,957,131	612,752	20.72
NE5	14.6	Petrous bone	1,464,369	1,101,369	75.21
NE6	14.5	Molar roots	3,050,993	243,718	7.99
NE6	14.4	Molar crown	1,711,503	90,957	5.31
NE6	14.2	Petrous bone	979,923	837,206	85.44
NE6	14.3	Temporal bone	814,829	504,755	61.95
BR2	8.5	Rib fragment	N/A	N/A	N/A
BR2	8.4	Petrous bone	1,203,775	681,149	56.58
IR1	10.2	Rib fragment	981,367	2,507	0.26
IR1	10.1	Petrous bone	1,867,212	872,472	46.73

Supplementary Table 3. Alignment statistics for all petrous bone samples sequenced on the Illumina Hiseq 2000 platform. Information about DNA concentration, measured using Invitrogen Qubit., and amplification is also provided.

Individual	Bone Powder (g)	DNA concentration (ng/µl)	Index sequence	PCR cycles	Mean Coverage	Total Reads	Aligned Reads (no duplicates)	Human nuclear DNA (%)
**KO1**	0.30	1.92	CCAACCT	10	1.24	89,112,108	55,958,647	62.8
**KO2**	0.32	0.99	TGCGTCC	12	0.13	85,439,425	8,655,489	10.13
**NE1**	0.35	3.17	TTCGCAA	12	22.12	1,106,919,660	961,367,273	86.85
**NE2**	0.35	2.49	CATCCGG	12	0.19	18,883,517	8,658,376	45.85
**NE3**	0.24	0.41	ATGCCGC	12	0.13	20,482,358	7,700,615	37.6
**NE4**	0.35	2.49	CGCGCAG	8	0.10	31,401,296	4,760,801	15.16
**NE5**	0.33	0.91	GATCTCG	10	1.04	78,344,674	55,639,944	71.02
**NE6**	0.30	1.65	GAATCTC	10	1.18	73,617,480	59,160,385	80.36
**NE7**	0.30	0.99	TTCGCAA	12	1.14	92,257,606	57,949,511	62.81
**CO1**	0.31	2.91	AATTCAA	10	1.13	181,628,477	62,787,334	34.57
**BR1**	0.30	1.26	CGCGCAG	12	0.81	58,622,526	41,534,364	70.85
**BR2**	0.39	2.11	TGGAATA	8	21.25	1,553,276,282	859,042,366	55.31
**IR1**	0.35	9.56	AATACCT	12	1.31	113,925,522	64,218,703	56.37

PCR, polymerase chain reaction.

Supplementary Table 4. HGDP+ modern populations. This dataset has been used for principal component analysis, admixture analysis and evaluating runs of homozygosity.

Region	Population	Code	Reference	Number of Individuals
**Caucasus**	Adygei	Ady	Li et al. 2008	17
**Caucasus**	Armenians	Arm	Behar et al. 2010, Yunusbayev et al. 2012	35
**Caucasus**	Georgians	Geo	Behar et al. 2010	20
**Caucasus**	Kuban Nogays	Nog	Yunusbayev et al. 2012	16
**Near East**	Bedouins	Bed	Li et al. 2008	48
**Near East**	Cypriotes	Cyp	Behar et al. 2010	12
**Near East**	Druze	Dru	Li et al. 2008	47
**Near East**	Jordanians	Jor	Behar et al. 2010	20
**Near East**	Lebanese	Leb	Behar et al. 2010	7
**Near East**	Palestinians	Pal	Li et al. 2008	51
**Near East**	Syrians	Syr	Behar et al. 2010	16
**Near East**	Turks	Tur	Behar et al. 2010	19
**Eastern Europe**	Belorussians	Bel	Behar et al. 2010	9
**Eastern Europe**	Bulgarians	Bul	Yunusbayev et al. 2012	13
**Eastern Europe**	Lithuanians	Lit	Behar et al. 2010	10
**Eastern Europe**	Romanians	Rom	Behar et al. 2010	16
**Eastern Europe**	Russians	Rus	Li et al. 2008, Behar et al. 2010	27
**Eastern Europe**	Ukranians	Ukr	Yunusbayev et al. 2012	20
**Eastern Europe**	Hungarians	Hun	Behar et al. 2010	20
**Western Europe**	Basque	Bas	Li et al. 2008	24
**Western Europe**	French	Fre	Li et al. 2008	29
**Western Europe**	Italians	Ita	Li et al. 2008	13
**Western Europe**	Orcadians	Orc	Li et al. 2008	16
**Western Europe**	Sardinians	Sar	Li et al. 2008	28
**Western Europe**	Spaniards	Spa	Behar et al. 2010	12
**Western Europe**	Tuscans	Tus	Li et al. 2008	8
**TOTAL**				**553**

Supplementary Table 5. Number of SNPs overlapping with the HGDP+ dataset called per ancient sample.

Individual	Number of SNPs
**KO1**	304,700
**KO2**	39,761
**NE1**	399,782
**NE2**	67,963
**NE3**	41,507
**NE4**	34,271
**NE5**	263,444
**NE6**	295,143
**NE7**	289,041
**CO1**	278,272
**BR1**	228,486
**BR2**	425,695
**IR1**	304,811

Supplementary Table 6. Negative control analysis results. Fifty base pair (bp) single-end sequencing was used. Adapters were trimmed using cutadapt as in **Supplementary Method 3.2**, retaining sequences greater than 16bp in length. Sequences were aligned to the GRCh37 build of the human genome with the mitochondrial sequence replaced by the revised Cambridge reference sequence and duplicates were removed as outlined in **Supplementary Method 3.2**. For each sample/control the number of reads aligning to the human genome was normalized by the total number of reads obtained in the MiSeq run. The contamination percentage was calculated as outlined in Supplementary Method **3.2**.

ID	Description	Normalized human reads	Contamination (%)	ID	Description	Normalized human reads	Contamination (%)
**8.1 - NE2**	**Sample**	**384,888**	**0.021**	**8.4 - BR2**	**Sample**	**167,576**	**0.048**
CG_8EC	Extraction Control	35	0.009	CG_8EC	Extraction Control	35	0.021
CG_8AC	Air Control	19	0.005	CG_8AC	Air Control	19	0.011
CG_LC1b	Library Control	19	0.005	CG_LC1b	Library Control	19	0.011
CG_LC2b	Library Control	8	0.002	CG_LC2b	Library Control	8	0.005
CG_C-1b	PCR Control	0	0	CG_C-1b	PCR Control	0	0
CG_C-2b	PCR Control	0	0	CG_C-2b	PCR Control	0	0
CG_C-4b	PCR Control	0	0	CG_C-4b	PCR Control	0	0
**10.1 - IR1**	**Sample**	**580,843**	**0.002**	**10.3 - NE1**	**Sample**	**583,410**	**0**
CG_10EC	Extraction Control	12	0.001	CG_10EC	Extraction Control	12	0
CG_LC1b	Library Control	19	0.001	CG_LC1b	Library Control	19	0
CG_LC2b	Library Control	8	0.001	CG_LC2b	Library Control	8	0
CG_C-1b	PCR Control	0	0	CG_C-1b	PCR Control	0	0
CG_C-2b	PCR Control	0	0	CG_C-2b	PCR Control	0	0
CG_C-4b	PCR Control	0	0	CG_C-4b	PCR Control	0	0
**10.5 - NE4**	**Sample**	**21,872**	**0.179**	**14.1 - KO2**	**Sample**	**18,319**	**0.502**
CG_10EC	Extraction Control	12	0.055	CG_14EC	Extraction Control	28	0.153
CG_LC1b	Library Control	19	0.087	CG_14AC	Air Control	46	0.251
CG_LC2b	Library Control	8	0.037	CG_LC1d	Library Control	8	0.044
CG_C-1b	PCR Control	0	0	CG_LC2d	Library Control	10	0.055
CG_C-2b	PCR Control	0	0	CG_C-1d	PCR Control	0	0
CG_C-4b	PCR Control	0	0	CG_C-2d	PCR Control	0	0
**14.2 - NE6**	**Sample**	**272,416**	**0.045**	**14.6 - NE5**	**Sample**	**359,985**	**0.034**
CG_14EC	Extraction Control	38	0.014	CG_14EC	Extraction Control	38	0.011
CG_14AC	Air Control	61	0.022	CG_14AC	Air Control	61	0.017
CG_LC1d	Library Control	11	0.004	CG_LC1d	Library Control	11	0.003
CG_LC2d	Library Control	13	0.005	CG_LC2d	Library Control	13	0.004
CG_C-1d	PCR Control	0	0	CG_C-1d	PCR Control	0	0
CG_C-2d	PCR Control	0	0	CG_C-2d	PCR Control	0	0
**16.1 - NE7**	**Sample**	**149,530**	**0.207**	**16.2 - CO1**	**Sample**	**136,772**	**0.226**
16EC	Extraction Control	282	0.189	16EC	Extraction Control	282	0.206
LC1_45	Library Control	16	0.011	LC1_45	Library Control	16	0.012
PCR1_46	PCR Control	5	0.003	PCR1_46	PCR Control	5	0.004
PCR2_47	PCR Control	7	0.005	PCR2_47	PCR Control	7	0.005
**6.4 - NE3**	**Sample**	**38,685**	**0.372**	**18.1 - KO1**	**Sample**	**379,663**	**0.055**
CG_6EC	Extraction Control	43	0.111	EC1_80	Extraction Control	9	0.002
CG_5ECb	Extraction Control	40	0.103	EC2_60	Extraction Control	29	0.008
CG_5ACb	Air Control	34	0.088	LC1_61	Library Control	19	0.005
CG_LC1b	Library Control	19	0.049	LC2_62	Library Control	61	0.016
CG_LC2b	Library Control	8	0.021	PCR1_63	PCR Control	93	0.024
CG_C-1b	PCR Control	0	0	PCR2_64	PCR Control	0	0
CG_C-2b	PCR Control	0	0	**16.3 - BR1**	**Sample**	**302,736**	**0.102**
CG_C-4b	PCR Control	0	0	16EC	Extraction Control	282	0.093
				LC1_45	Library Control	16	0.005
				PCR1_46	PCR Control	5	0.002
				PCR2_47	PCR Control	7	0.002

Supplementary Table 7. Mean read length and standard deviation per sample. Calculated on reads aligning to the human nuclear genome. Length was calculated using MapDamage 2.0 as outlined in 4.3. Reads considered had a minimum length of 30 bp and a maximum length of 100 bp.

Individual	Mean Length	Standard Deviation
**KO1**	70.05	21.22
**KO2**	53.36	15.23
**NE1**	68.65	20.91
**NE2**	69.58	20.86
**NE3**	57.72	20.49
**NE4**	65.26	19.32
**NE5**	60.18	19.9
**NE6**	63.84	21.19
**NE7**	63.47	21.62
**BR1**	61.74	20.02
**BR2**	70.48	21.16
**CO1**	57.99	18.67
**IR1**	65.46	21.7

Supplementary Table 8. Mitochondrial contamination estimates. Calculated as mean percentage of mismatch depth over the total depth at each position.

Individual	Mean Coverage mtDNA	Number of haplotype defining positions	% C + MD	% C
**KO1**	69.5	31	0.13	0.15
**KO2**	8.29	31	0.52	0.81
**NE1**	183.78	30	0.11	0.03
**NE2**	12.39	34	0.04	0.00
**NE3**	8.88	37	0.48	0.54
**NE4**	5.35	19	0.00	0.00
**NE5**	55.55	33	0.10	0.10
**NE6**	73.88	34	0.11	0.09
**NE7**	70.31	37	0.09	0.07
**CO1**	47.03	29	0.13	0.00
**BR1**	47.26	33	0.12	0.08
**BR2**	185.08	35	0.07	0.04
**IR1**	80.42	38	0.09	0.06

mtDNA, mitochondrial DNA; % C, percentage contamination excluding sites with possible molecular damage; % C+MD, percentage contamination including possible molecular damage.

Supplementary Table 9. Test 1 and Test 2 performed as in [91]. . Number of primary (P) and secondary (S) alleles in ancient male samples at X chromosome sites found to be polymorphic in European populations (0) and adjacent sites 4 bases upstream and downstream.

Sample	Test	Allele	-4	-3	-2	-1	0	1	2	3	4
**KO1**	**1**	P	22,692	22,673	22,703	22,569	22,575	22,591	22,604	22,816	22,717
		S	87	91	89	114	174	102	87	69	108
**KO1**	**2**	P	7,163	7,156	7,171	7,130	7,138	7,121	7,133	7,197	7,183
		S	25	28	25	39	47	37	27	24	26
**NE5**	**1**	P	11,703	11,756	11,580	11,589	11,664	11,636	11,680	11,676	11,681
		S	63	61	65	67	100	72	42	56	56
**NE5**	**2**	P	3,720	3,738	3,677	3,681	3,705	3,700	3,717	3,715	3,723
		S	20	22	22	22	35	20	12	18	14
**NE6**	**1**	P	18,291	18,215	18,336	18,241	18,309	18,388	18,430	18,424	18,470
		S	73	79	65	102	144	96	76	71	69
**NE6**	**2**	P	5,804	5,769	5,815	5,779	5,812	5,830	5,835	5,827	5,846
		S	20	27	21	38	39	27	24	24	17
**NE7**	**1**	P	17,637	17,620	17,598	17,623	17,582	17,686	17,766	17,765	17,726
		S	57	66	60	86	136	82	78	71	64
**NE7**	**2**	P	5,592	5,583	5,580	5,575	5,563	5,596	5,622	5,616	5,610
		S	22	25	15	40	49	29	21	25	22
**BR2**	**1**	P	3,654,337	3,653,258	3,649,463	3,642,580	3,632,505	3,641,908	3,648,359	3,651,768	3,653,478
		S	14907	14529	14708	17628	26461	17593	14855	14605	14972
**BR2**	**2**	P	333,185	333,265	333,217	332,787	331,933	332,798	333,080	333,117	333,196
		S	1,393	1,396	1,311	1,666	2,462	1,614	1,390	1,409	1,389
**IR1**	**1**	P	26,579	26,499	26,425	26,533	26,462	26,421	26,706	26,772	26,808
		S	117	103	95	131	222	118	96	86	94
**IR1**	**2**	P	8,344	8,318	8,298	8,330	8,320	8,315	8,396	8,417	8,425
		S	35	32	29	45	70	36	34	26	30

Supplementary Table 10. X chromosome contingency table for ancient male samples. Contingency table was prepared as in [91]..

Sample	Test		Number of Alleles at Polymorphic sites	Average of the Number of Alleles at 8 Adjacent Sites
**KO1**	**1**	P	22575	22,670.63
		S	174	93.375
		e	0.007707641	0.004
**KO1**	**2**	P	7138	7,156.75
		S	47	28.875
		e	0.006584477	0.004
**NE5**	**1**	P	11664	11,662.63
		S	100	60.25
		e	0.008573388	0.005
**NE5**	**2**	P	3705	3,708.88
		S	35	18.75
		e	0.009446694	0.005
**NE6**	**1**	P	18309	16,082.00
		S	144	66.125
		e	0.007864984	0.004
**NE6**	**2**	P	5812	5,813.13
		S	39	24.75
		e	0.006710255	0.004
**NE7**	**1**	P	17582	17,677.63
		S	136	70.5
		e	0.007735184	0.004
**NE7**	**2**	P	5563	5,596.75
		S	49	24.875
		e	0.008808197	0.004
**BR2**	**1**	P	3632505	3,649,393.88
		S	26461	15,474.63
		e	0.007284505	0.004
**BR2**	**2**	P	331933	333,080.63
		S	2462	1,446.00
		e	0.007417159	0.004
**IR1**	**1**	P	26462	26,592.88
		S	222	105
		e	0.008389389	0.004
**IR1**	**2**	P	8330	8,355.38
		S	70	33.375
		e	0.008403361	0.004

P, number of primary alleles; S, number of secondary alleles; e, observed probability error.

Supplementary Table 11. Contamination estimations based on Test 1 and Test 2 [91]..

Sample	Test	Contamination (%)	P-value*
**KO1**	1	0.66	8.26E-07
**KO1**	2	0.21	3.90E-02
**NE5**	1	0.67	1.89E-03
**NE5**	2	1.2	3.95E-02
**NE6**	1	0.53	7.84E-06
**NE6**	2	0.71	1.02E-01
**NE7**	1	0.71	5.12E-06
**NE7**	2	0.42	5.05E-03
**BR2**	1	0.26	< 2.2e-16
**BR2**	2	0.29	< 2.2e-16
**IR1**	1	1.05	5.69E-11
**IR1**	2	0.81	5.14E-04

* Obtained using Fisher's exact test.

Supplementary Table 12. Mitochondrial DNA haplotypes and haplogroups for all ancient samples. Haplogroup assignment quality was calculated using Haplogrep [95]. Expected mutations for each haplogroup are in bold.

Individual	Mean Coverage	Haplotype	Haplogroup	Quality (%)
**KO1**	69.5	**73G, 150T, 241G, 263G,** 310C^, 315.1C*, 513.1CGCAº, **750G, 1095C, 1391C, 1438G,** 1719A^, **2706G,** 3483A^, **4769G, 6683T, 7028T, 8860G, 11122A, 11151T, 11719A, 12245C,** 12397G^, **13167G, 13359A, 14239T, 14766T, 15326G, 15940C, 16311C,** 16519C*	R3	93
**KO2**	8.29	**73G**, 114T^, **263G,** 309.1C*, **750G, 1189C, 1438G, 1811G, 2706G, 3480G, 4769G,** 6239Aº, **7028T, 8860G, 9055A, 9698C, 10398G,** 10542Tº, **10550G, 11299C, 11467G, 11719A,** 11962Gº, **12308G, 14167T, 14766T, 14798C, 15326G**, 16093C^, **16224C**, 16519C*	K1	95.6
**NE1**	183.78	**73G, 150T, 263G,** 309.1C*, 310C^, **723G, 750G, 960.1C, 1438G, 1721T, 2706G, 3197C, 4769G, 7028T, 7768G, 8860G, 9477A, 11467G, 11719A, 12308G, 12372A, 13017G, 13617C, 13637G, 14182C, 14766T, 15326G,** 16189C^, **16270T**	U5b2c	96.7
**NE2**	12.39	**263G**, 315.1C*, **750G, 1438G, 4769G, 8860G, 15326G,** 16266T^, 16519C*	H	96
**NE3**	8.88	**73G, 153G, 195C, 263G, 750G, 1438G, 1719A, 2706G, 4769G, 6371T, 7028T, 8393T, 8860G, 11719A, 12705T,** 13134G, **13708A, 13966G, 14470C, 14766T, 15326G, 15927A,** 16519C*	X2b	92.6
**NE4**	5.35	**73G, 185A, 228A, 263G, 295T, 750G, 2706G, 3010A, 4216C, 4769G, 7028T, 8860G, 10398G, 11251G, 11719A, 14766T, 15326G, 16069T, 16126C**	J1c	86.5
**NE5**	55.55	**73G, 185A, 228A, 263G, 295T,** 309.1C*, **462T, 482C, 489C, 750G, 1438G,** 1462A^, **2706G, 3010A, 3394C, 4216C, 4769G, 7028T, 8860G, 10398G, 11251G, 11719A, 12612G, 13708A, 14766T, 14798C, 15326G, 15452A, 16069T, 16126C**	J1c1	98.6
**NE6**	73.88	**73G, 263G,** 309.1C*, 310C^, **497T, 750G, 1189C, 1438G, 1811G, 2706G, 3480G, 4769G, 7028T, 7559G, 8860G, 9055A, 9698C, 10398G, 10550G, 11299C, 11467G, 11719A, 12308G, 12372A, 13117G, 13590A, 14167T, 14766T, 14798C, 15326G,** 16093C^, **16224C, 16311C,** 16519C*	K1a3a3	98.1
**NE7**	70.31	**73G, 152C, 199C, 204C,** 207A^, **263G,** 315.1C*, **669C, 750G, 1438G, 1719A, 2702A, 2706G, 3336C, 4769G, 5315G, 7028T,** 7624C^, **8860G, 8901G, 10238C, 10398G, 11719A, 12501A, 12705T, 13780G, 14766T, 15043A, 15326G,** 16086C^, 16147A^, **16172C, 16223T, 16248T, 16320T, 16355T,** 16519C*	N1a1a1a	90.8
**CO1**	47.03	**263G, 750G, 1438G, 4769G, 8860G, 15326G,** 16519C*	H	100
**BR1**	47.26	**73G, 146C, 152C, 263G,** 315.1C*, **498del, 750G, 1189C, 1438G, 1811G, 2706G, 3480G, 4769G, 7028T, 8860G, 9055A, 9093G, 9698C, 10398G, 10550G, 11299C, 11377A, 11467G, 11719A, 12308G, 12372A, 14167T, 14766T, 14798C, 15326G, 16224C, 16311C,** 16519C*	K1c1	100
**BR2**	185.08	**73G, 114T, 263G,** 315.1C*, **497T, 750G, 1189C, 1438G, 1811G, 2706G, 3480G, 3552C, 4769G, 7028T, 8860G, 9055A,** 9617G^, **9698C, 10398G, 10550G, 11299C, 11467G, 11719A, 11914A, 12308G, 12372A, 14167T, 14766T, 14798C, 15326G, 16093C,** 16172C^, **16224C, 16311C,** 16519C*	K1a1a	97.4
**IR1**	80.42	**73G,** 152C^, **263G, 489C, 709A, 750G, 1438G, 2706G, 4769G, 4833G, 5108C, 5601T, 7028T, 7600A,** 8547C^, **8701G, 8860G, 9377G, 9540C, 9575A, 10398G, 10400T, 10873C, 11719A, 12705T, 13563G, 14200C, 14569A,** 14587G^, **14766T, 14783C, 15043A, 15301A, 15326G, 16223T, 16278T, 16362C,** 16519C*	G2a1	95.5

* hotspot; ^ local private mutation; º global private mutation; all as defined by the online tool Haplogrep.

Supplementary Table 13. Y-chromosome haplogroup assignment in all ancient males.

Final assigned haplogroup is highlighted in bold.

Individual	Position (GRCh37)	Reference Allele	Alternate Allele	Allele in Ancient Sample	Associated Haplogroup	Depth of coverage
**KO1**	8590752	C	T	T	IJ	2
	14144593	A	G	G	IJ	1
	14484379	A	C	C	I	1
	14847792	A	C	C	I	1
	15023364	T	C	C	I	1
	16354708	G	A	A	I	1
	19048602	G	A	A	I	1
	16638804	A	G	G	I2	4
	18700150	C	T	T	I2	2
	7879415	A	C	C	**I2a**	1
**NE5**	2756519	C	T	C	BT	1
	14079528	T	C	T	BT	1
	22748506	G	T	G	BT	1
	7629098	T	C	C	C	1
	14924643	G	T	T	C	1
	17947542	C	T	T	C	1
	6845955	G	A	A	**C6**	1
**NE6**	6908261	G	A	G	BT	1
	6932831	A	G	A	BT	1
	7645425	G	A	G	BT	1
	21674378	T	C	T	BT	1
	21938158	A	C	A	BT	1
	22228683	G	A	G	BT	1
	14924643	G	T	T	C	1
	15437564	C	T	T	C	1
	7629098	T	C	C	C	2
	14263271	G	A	A	C	1
	14263293	C	T	T	**C6**	1
**NE7**	6753519	G	A	G	IJK	2
	15023364	T	C	C	I	2
	19038302	A	C	C	IJ	1
	19166861	T	C	C	IJ	1
	7879415	A	C	C	**I2a**	1
**BR2**	6753519	G	A	G	IJK	9
	7173143	A	G	A	IJK	13
	8590752	C	T	T	IJ	15
	14144593	A	G	G	IJ	10
	19038302	A	C	C	IJ	13
	19166861	T	C	C	IJ	10
	14237131	C	T	T	J	9
	22749853	A	C	C	J	6
	19179335	T	C	C	J	14
	14969634	T	G	G	J2	7
	22243566	C	T	T	J2a	6
	22942897	T	C	C	**J2a1**	10
**IR1**	6753519	G	A	G	IJK	1
	15472863	T	C	T	K	1
	20837553	T	C	T	K	1
	23550924	C	A	C	K(xLT)	2
	19279765	A	G	G	NO	1
	22214221	T	G	G	NO	1
	22665262	A	G	G	NO	1
	15469724	G	A	A	**N**	2

Supplementary Table 14. Comparison between observed and imputed data for computing the ROH.

Sample & Description	Number of segments	Total ROH (Mb)	Average length (kb)	Percentile
**NE1 0.5x imputed**	152	126.322	831.066	82
**NE1 1x imputed**	146	123.106	843.194	81
**NE1 2.5x imputed**	149	125.658	843.345	82
**NE1 20x observed**	152	127.691	840.075	82
**BR2 0.5x imputed**	127	103.9	818.11	67
**BR2 1x imputed**	128	104.558	816.861	68
**BR2 2.5x imputed**	128	104.138	813.579	68
**BR2 20x observed**	129	106.086	822.376	69

Mb, megabase; kb, kilobase; ROH, runs of homozygosity.

Supplementary Table 15. Genotyping results for the 8-plex system. Observed genotypes are reported for NE1 and BR2 while imputed genotypes are presented for the remaining 11 samples. Position and reference allele are with reference to the GRCh37 build of the human genome.

**Chr**	**5**	**6**	**14**	**15**	**15**	**15**	**16**	**20**	
**SNP**	**rs16891982**	**rs12203592**	**rs12896399**	**rs1545397**	**rs12913832**	**rs1426654**	**rs885479**	**rs6119471**	**Skin colour**
**Reference**	**C**	**C**	**G**	**A**	**A**	**A**	**G**	**C**	
**Alternate**	**G**	**T**	**T**	**T**	**G**	**G**	**A**	**G**	
**KO1**	CC	CC	GT	AA	GG	AG	GG*	CC	Inconclusive
**KO2**	CG*	CC	GG	AA	AG*	AG*	GG*	CC*	Inconclusive
**NE1**	CC	CT	TT	AA	AG	AG	GG	CC	Inconclusive
**NE2**	CC	CC*	GT	AT	AA*	AA	GG*	CC*	Inconclusive
**NE3**	CG*	CC	GG*	AA	AG*	AA*	GA*	CC	Inconclusive
**NE4**	CG*	CC	GG	AA	GG*	AA	GG*	CC	Light skin
**NE5**	CC*	CC	GT	AA	AG	AA*	GA*	CC	Inconclusive
**NE6**	CC	CC	GG	AA	GG	AA	GG	CC	Light skin
**NE7**	CG	CC	GG	AA	GG*	AA	GG	CC	Light skin
**CO1**	GG	CC	GT	AA	AG	AA	AA*	CC	Light skin
**BR1**	CG	CT*	GG*	AA	AG*	AA	GG	CC	Inconclusive
**BR2**	GG	CT	GG	AA	AG	AA	GG	CC	Light skin
**IR1**	GG	CC	TT	AA	AG*	AA	GG	CC	Light skin

* Genotypes with imputed probabilities below 0.85; Chr, chromosome; SNP, single nucleotide polymorphism

Supplementary Table 16. Hirisplex system results. Phenotype prediction and associated probabilities obtained for hair color and shade as well as eye color using the 24 SNPs included in the Hirisplex system.

Identifier	Hair Colour and Shade Prediction	Associated Probability					Eye Colour Prediction	Associated Probability		
		Red	Black	Blond	Light	Dark		Blue	Intermediate	Brown
**KO1**	**Black/dark brown**	0	0.612	0.092	0.07	0.93	**Blue**	0.5	0.125	0.38
**KO2**	**Dark brown/ light brown**	0	0.655	0.104	0.12	0.89	**Brown**	0.02	0.083	0.892
**NE1**	**Black/dark brown**	0	0.802	0.013	0.03	0.97	**Brown**	0.03	0.081	0.892
**NE2**	**Black/dark brown**	0	0.863	0.003	0	1	**Brown**	0	0.006	0.994
**NE3**	**Black/Dark brown**	0	0.581	0.063	0.07	0.93	**Brown**	0.02	0.083	0.892
**NE4**	**Black/dark brown**	0	0.829	0.017	0.02	0.98	**Brown**	0	0.013	0.987
**NE5**	**Dark brown/ light brown**	0	0.622	0.069	0.12	0.88	**Brown**	0.01	0.043	0.948
**NE6**	**Dark brown/ light brown**	0	0.49	0.188	0.32	0.68	**Intermediate**	0.37	0.156	0.477
**NE7**	**Light brown/ dark blond**	0	0.176	0.63	0.79	0.21	**Blue**	0.79	0.138	0.071
**CO1**	**Dark brown/ light brown**	0	0.443	0.317	0.55	0.45	**Brown**	0.21	0.161	0.632
**BR1**	**Black/dark brown**	0	0.748	0.025	0.03	0.97	**Brown**	0.04	0.151	0.81
**BR2**	**Dark brown/ light brown**	0	0.327	0.235	0.46	0.54	**Brown**	0.14	0.241	0.62
**IR1**	**Light brown/ dark blond**	0	0.326	0.443	0.6	0.41	**Brown**	0.31	0.142	0.552

Supplementary Table 17. Genotyping results for the Hirisplex system. Observed genotypes are reported for NE1 and BR2 while imputed genotypes are presented for the remaining 11 samples. Reference allele is with reference to the GRCh37 build of the human genome.

SNP	code	Ref	Alt	KO1	KO2	NE1	NE2	NE3	NE4	NE5	NE6	NE7	CO1	BR1	BR2	IR1
N29insA^	-	-	A	-	-	-	-	-	-	-	-	-	-	-	-	-
rs11547464	-	G	A	GG	GG	GG	GG	GG	GG	GG	GG	GG	GG	GG	GG	GG
rs885479	f	G	A	GG*	GG*	GG	GG*	GA*	GG*	GA*	GG	GG	AA*	GG	GG	GG
rs1805008	-	C	T	CC	CC	CC	CC	CC	CC	CC	CC	CC	CC	CC	CC	CC
rs1805005	-	G	T	GG	GG*	GG	GG	GG	GG*	GG	GG	GG	GG	GG*	GG	GG
rs1805006	-	C	A	CC	CC	CC	CC	CC	CC	CC	CC	CC*	CC	CC	CC	CC
rs1805007	-	C	T	CC	CC	CC	CC	CC	CC	CC	CC	CC	CC	CC	CC	CC
rs1805009	-	G	C	GG	GG	GG	GG	GG	GG	GG	GG	GG	GG	GG	GG	GG
Y152OCH^	-	C	A	CC	CC	CC	CC	CC	CC	CC	CC	CC	CC	CC	CC	CC
rs2228479	-	G	A	GG	GG	GG	GG	GG	GG	GG	GG	GG	GG	GG	GG	GG
rs1110400	-	T	C	TT	TT	TT	TT	TT	TT	TT	TT	TT	TT	TT	TT	TT
rs28777	s	C	A	CC	CA*	CA	CC	CA*	CA*	CA	CA	CA	AA	CA	AA	AA
rs16891982	s	C	G	CC	CG*	CC	CC	CG*	CG*	CC*	CC	CG	GG	CG	GG	GG
rs12821256	f	T	C	TT	TT	TT	TT	TT	TT	TC	TT	TT	TT	TT	TC	TT
rs4959270	-	C	A	CC	CC	AA	CA*	CA*	CC*	CA	CA	CA	CC	CA	AA	CC
rs12203592	-	C	T	CC	CC	CT	CT*	CC	CC	CC	CC	CC	CC	CT*	CT	CC
rs1042602	f	C	A	CC*	CC*	CC	CA*	CA*	CC*	CA	CC*	CC	CC*	CC	CC	CC
rs1800407	f	C	T	CC	CC	CC	CC	CC	CC	CC	CC	CT*	CC	CC	CC	CC
rs2402130	s	G	A	GA	AA	AA	GA	GG	GA	AA	GA	AA	AA	GA	GA	AA
rs12913832	fs	A	G	GG	AG*	AG	AA*	AG*	GG*	AG	GG	GG*	AG	AG*	AG	AG*
rs2378249	fs	G	A	GA	AA	AA	GA*	GA*	AA	AA	AA	TT	AA	AA	AA	AA
rs12896399	s	G	T	GT	GG	TT	GT	GG*	GG	GT	GG	GG	GT	GG*	GG	TT
rs1393350	f	G	A	GG*	GG	GG	GG	GG	GG*	GA	GG	GG	GA	GG*	GG	GA
rs683	fs	C	A	CA	CC	CA	CC*	CA	CA*	CC	CC*	AA	AA	AA	CA	CA

* Genotypes with imputed probabilities below 0.85; ^ Un-imputed genotypes, the ancestral allele was considered instead; - no differences to alleles reported in [157]; s complementary alleles to those reported in [157]; f reference and alternate allele flipped between genotypes in ancient samples and those reported in [157]; SNP, single nucleotide polymorphism; Ref., reference; Alt., alternate.

SUPPLEMENTARY NOTES

1 - Archaeological context and Samples Information

The Great Hungarian Plain (**Supplementary Fig. 1**) is a major lowland in the Carpathian basin area which is connected with the Mediterranean, the Pontic steppe, and the loess-lands of central Europe [14,43]. This area represents a crossroads of human prehistoric cultures.

The earliest agricultural communities belong to the Körös culture (Criş in Romania), which is part of the Balkan Early Neolithic complex, the “First Temperate Neolithic”[10–12]. Early Neolithic burials across eastern Europe are characterized by a lack of cemeteries, in contrast to those of later Neolithic periods. Furthermore, the majority of burials are found inside what seem to be refuse pits. In most cases it is not possible to discern whether the artifacts recovered from these pits were simply deposited or discarded into the pit or were rather interred with the person as grave goods [44,45]. In the specific case of skeletons from Körös culture sites from Hungary only 13 of the 184 burials were accompanied by objects or fragments of objects that could be identified to belong to a grave assemblage [46].

Around 5,600/5,500 cal. BC (calibrated years BC) major changes took place in the Carpathian Basin. In the Great Hungarian Plain the Körös culture was followed by the Linearbandkeramik (LBK) cultural complex, which consisted of two synchronous regional groups: the Alföld Linear Pottery (ALP) culture - also known in the literature as Alföld Linearbandkeramik Culture and Alföldi Vonaldíszes Kerámia (AVK) [14,15] - in the Great Hungarian Plain and the Eastern LBK variant in west Hungary [15]. The Western LBK group dispersed agriculture into the Central European loess belt and became the dominant LBK culture of Europe. During the period between 5,500–5,000 BC ALP presence in the Hungarian Plains expanded into the Tisza lake region (eastern Hungary) and was accompanied by a synchronous differentiation into local groups. Archaeological research indicates that in this region the Late Neolithic (ca. 5,000-4,500 BC) comprises the tell settlement culture of the Tisza–Herpály–Csőszhalom complex which is contemporaneous with the Lengyel Culture of Transdanubia. These two cultural complexes met in the foreland of the northern Hungarian Range. This cultural division had its roots in the Middle Neolithic development.

In the last phase of the Neolithic, around 4,500 BC, at the end of the Atlantic Period, the climate favorable for crop cultivation was replaced by a cooler sub-boreal climate. This change had a major impact on the economy of Late Neolithic societies, and resulted in a shift from crop agriculture to an increased focus on animal husbandry. In the course of complex processes new network-systems emerged with the formation of the Copper Age cultural entities on the Great Hungarian Plain [47]. While there is no apparent discontinuity in the cultural sequences of the Late Neolithic and Early Copper age, there is change in settlement structures and burial customs, which correspond to differences in social status and wealth [48]. The Copper Age period in the Great Hungarian Plain was divided traditionally into 3 phases: Tiszapolgár, Bodrogkeresztúr/Hunyadihalom and Baden. Based on radiocarbon dates the following chronological framework was proposed: Early Copper Age (4,500/4,400–4,000 BC), Middle Copper Age (4,000–3,600/3,500 BC), and Late Copper Age (3,600/3,500–2,600/2,500 BC) [49,50].

During the period from the end of the fourth to the middle of third millennium BC North Pontic kurgans (burial mounds), characteristic for steppe, became distributed into the Tisza region. Supporting the archaeological evidence, isotopic analyses also indicate the appearance of migrant groups [51]. During the course of the Bronze Age between 2,700/2,500 and 900/800 tell settlements occurred repeatedly and with the enclosured sites, hillforts represented special cultural attitudes of the new socio-political entities. The deposition of metal “hoards” in special places - a general phenomenon of European Bronze and Iron Age societies - was a new custom of these times [52].

Iron metallurgy first appeared in central Europe during the 1st millennium BC. During the early phase of the Iron Age the regions east and west of the Danube were parts of two separate cultural provinces [53]. The Eastern variant of the Central European Hallstatt culture prevailed in Transdanubia, while pre-Scythian, and later Scythian, cultures inhabited the Great Hungarian Plain and the northern mountainous regions. Despite decades of extensive excavations there are only a handful of Iron Age settlements on the Great Hungarian Plain. This is due to the fact that the pre-Scythian populations practiced a form of nomadic stockbreeding and their transient settlements left few traces in the archaeological record [54]. The origin of the pre-Scythian populations of the Great Hungarian Plain (the so called ‘Mezőcsát communities’) remains unclear. The excavation of Early Iron Age burials from this region has provided important new information as burial rites resembled the mortuary practices of the pre-Scythian period in the steppe, suggesting that the Mezőcsát communities were not descendants of the local Late Bronze Age population but most likely arrived to the Great Hungarian Plain from the East [54].

1.1 Archaeological sites

Tiszaszőlős-Domaháza is a small site at the northernmost settlement of the Körös culture along the Middle Tisza River. The Körös cultural phase includes a pit, a house plan and some graves [21]. The house plan was very regular in shape, forming an oblong of 12×5.5m. It is directed NW–SE along its longer axis and consists of a thick layer of ceramic and mussel shell fragments and, in the centre of its southern half, the burnt remains of a hearth. Apart from the Körös culture pit and house other later occupational phases were excavated at the site including early LBK, Early Bronze Age and Medieval phases.

A total of 7 individuals were exhumed from Neolithic contexts: two complete skeletons, two nearly complete skeletons identifiable by their dispersed body parts, and three single bones. One skeleton (grave 2–3), separated into two or three parts probably by later disturbance, was found in the lower 10–13th layers of Pit 6. The two most complete skeletons (Grave 1 and 6) were found in the eastern and northern sections of Pit 6. These belonged to regular burials in flexed positions. The other two sets of human remains (graves 4 and 5) were exhumed from the same pit: a skull (Grave 4, specimen **KO1,** recovered at 140-155 cm depth, **Supplementary Table 1**) and a mandible (Grave 5). Other human remains were found in the area of the house plan and belonged to two separate individuals (Grave 7 and 8). The skull of Grave 4 was found in clear Körös context in the lower part of Pit 6.

Radiocarbon dates provide a comprehensive chronological framework for the site. Graves 2, 3, 4 and 8 as well as the greatest part of the bones and grains date to Phase B of this site, 5,710–5,630 BC, i.e. the Körös Culture. The radiocarbon dates are thus

in agreement with the archaeological context and stratigraphy and suggest that the skull dates to the time span between ~5,800 and ~5,650 cal BC. Other graves and some of the faunal remains belong to later Neolithic phases of the Szatmár Group, ALBK, and the Szakálhát Group [55].

Berettyóújfalu-Morotva-Liget is situated in Hajdú-Bihar County, on the southern bank of Berettyó River. It contains a sequence of archaeological horizons spanning from Early Neolithic Körös to Late Medieval. The first excavation season in 1977 revealed 3 large waste pits (A, B and C), and 3 burials (2 adults from pit B and an infant from pit C) and human remains of 2 additional individuals from disturbed graves. All pits and graves included painted pottery and were dated to the Late ALP (Esztár Group) [56,57].
In 2011 an additional area of 1700 m^2 was excavated and pits, wells, ditches and postholes of the Neolithic Körös Culture were revealed. Grave 44 (specimen **KO2**, **Supplementary Table 1**) is of an adult male, which was excavated at the western border of the main area. The skeleton was laid at a N–S orientation on the left side in a flexed position with both arms folded and the hands placed in front of the face. The burial had no associated grave goods. The N-S orientation of the grave, the position of the skeleton, and the lack of grave goods are typical for the funerary practices of the Körös Culture [46,58].

Polgár-Ferenci hát is an ALP site, which is located on the former bank of the Tisza floodplain. A contiguous surface measuring approximately 4 ha was investigated during two seasons 2001/2002. The site consists of a section of an elongated oval double ditch system [59–62]. Traces of intensive human activity, including the remains of several burnt houses and a thick layer of refuse, were found within this enclosure.
A total of 113 burials were recovered. The graves were clustered in smaller and larger concentrations within the excavated settlement area. Their density is higher in the proximity of ditches and within the enclosure. The grave goods recovered included obsidian cores, beads, armrings made of spondylus shells and vessels containing red ochre. These point to long-range trade contacts. A special group of burials is represented by regular inhumation graves in which the skull was missing [62].
Recovered pottery found within the circular ditch system is of typical Middle Neolithic Tiszadob–Bükk–Esztár and Szakálhát–Vinča ceramic styles. Grave 325/500 (specimen **NE1**, **Supplementary Table 1**) is an oval pit, orientation SE–NW, of a child, which was laid on the left side in flexed position. Red ochre was found on the skull. The only grave good is a deep bowl with incised decoration, which was placed in front of the skull. Grave 839/1198 (specimen **NE4**, **Supplementary Table 1**) is an oval pit, orientation SE–NW, of a female laid on her left side in a crouched position. Again, red ochre was found on the skull. The only grave good was one small obsidian blade found near the skull.

Debrecen, Tócó-part, Erdőalja is situated in Hajdú-Bihar County on the northern periphery of Debrecen, eastern Hungary. Large-scale rescue excavations associated with the construction works of the M35 bypass road were carried out during 2008–2009. The site yielded Middle Neolithic ALP, Copper Age, Early Bronze Age (Makó culture), Late Iron Age (Celtic/La Tène culture), Roman imperial (Sarmatian), Late Avarian, and Early and Late Medieval cultures [63–65].
The ALP occupation is dated to the late phase of the ALP culture and includes ~50 extensive clay pits with various finds: broken pottery, fauna, stone tools and

fragments of wattle and daub. Some postholes, which were structural elements of the long rectangular houses, were excavated between the pits.
Thirteen mostly intact graves were excavated. The analyzed skeleton from Grave 1412-1761 (specimen **NE2**, **Supplementary Table 1**) was dug into the top of a big clay pit (304/749). The skeleton was on the left side in a crouched position in an E–W orientation. Some shards of painted pottery were placed in the southern part of the grave near the feet [64].

Garadna, Elkerülő út, 2 is a site located in the Hernád valley, northeast Hungary. During excavation an area of 2,000 m^2 was excavated revealing occupation phases dating from the Middle Neolithic Bükk culture to the Late Bronze Age. Thirteen Middle Neolithic graves were unearthed. Ten burials were found each in a single grave, one skeleton was from a disturbed context and two skeletons were from refuse pits. The graves (including the one of **NE3**, a juvenile, 4–6 years, **Supplementary Table 1**) contained various grave goods including vessels, grinding stones, and necklaces made from spondylus beads [66,67].

Apc-Berekalja I is situated at the easternmost periphery of the western LBK close to the ALP cultural limit near to the Zagyva valley at the border of Pest and Heves Counties. It is one of the largest LBK site in Hungary and while it was settled primarily during the Late LBK Musical Note and Zseliz/Želiezovce phases (~5,300-5,000 cal BC), the settlement was first occupied in the Early LBK phase between 5,400 and 5,300 cal BC. Several LBK houses, pits and graves were excavated as well as finds from the Late Copper Age, Early and Middle Bronze Age, Roman and Early Medieval Periods.
No radiocarbon results have been published from the site so far, though several analyses have been conducted, including some ^{14}C measurements in the Oxford, Mannheim and Poznan radiocarbon laboratories. The 16 graves date to the Middle and Late Neolithic, Copper Age and Bronze Age periods, as well as the Roman period.
Grave 6 (specimen **NE6**, **Supplementary Table 1**) contained a fragmentary juvenile skeleton laid on its right side in flexed position. It was found near a LBK house and was radiocarbon dated to the Zseliz/Želiezovce phase of the LBK. Grave 2 (specimen **NE7**, **Supplementary Table 1**) was of a juvenile skeleton positioned on its right side in a semi-flexed position. The grave goods included a ceramic bowl near its lower abdomen, containing animal bones. Some Spondylus beads were placed around the head and waist as ornaments. These grave goods and the radiocarbon date (~4,310-4,530 cal BC) imply that it belonged to the Late Neolithic Lengyel culture.
Grave 15 (specimen **CO1**, **Supplementary Table 1**) contained a skeleton of an adult female, which was buried in a semi-flexed position on its right side. Based on the radiocarbon date (~2,670-2,990 cal BC) it can be attributed to the late phase of the Copper Age Baden culture, which is present in several sections of the site.

Kompolt-Kígyósér is situated between Kál and Nagyút in Heves County, eastern Hungary. In 1994, an area of 24,000 m^2 was excavated yielding artifacts spanning from the Neolithic to Medieval times. The ALP cultural features include a series of long and beehive-shaped pits and six burials. The ceramic finds in the pits and graves belonged to the Late ALP with some Szakálhát and Bükk type pottery fragments.
Grave 17/7 (specimen **NE5**, **Supplementary Table 1**) contained no grave goods. The skeleton was in a fragmentary condition with many missing bones. It was positioned

on its left side in flexed position. Its chronological attribution to the Late ALP is based on both the present radiocarbon date and another determination from the Debrecen laboratory.
Grave 7/3 (specimen **BR1**, **Supplementary Table 1**) contained a complete skeleton, which was laid on his left side in flexed position. The grave contains a small pot characteristic of the Neolithic ALP phase of the site [68]. However, the direct radiocarbon date (1977 -2194 cal. BC, 2SD, **Supplementary Table 1**) indicates that the specimen is from the Bronze Age. This also agrees with the presence of Makó culture artifacts in other locations in the site.

Ludas-Varjú-dűlő is situated north of Ludas at the foothills zone of the Mátra Mountain in Heves County. This large site area was excavated in 1998–2002, revealing a rich Late Bronze Age settlement area of 18 ha [69,70]. This settlement had a monumental round structure (a ditch bordered by a palisade), which probably served as a ceremonial center on its eastern side and had an urn cemetery at its northern part. The inner part of the settlement was full of pits, but house structures were not preserved.
According to the radiocarbon dates the Late Bronze Age settlement spans the period between 1,540–1,000 cal BC. More than twenty pits yielded human burials. Pit 2161 contained two skeletons: a 2–3 year old child (specimen **BR2**, **Supplementary Table 1**) and an adult female with a similar absolute date as the child (Deb11101, 3130±70 uncal. BP). The ceramics in the pit are typical for the Late Bronze Age Kyjatice Culture, which is also in good agreement with the absolute radiocarbon dates.

At the southern edge of the settlement a pre-Scythian cemetery was excavated that contained two groups of skeletons. The first, with 7 graves, was very poorly preserved, while the second (with 12 graves) was better preserved. Grave 2638 (specimen **IR1**, a 4-6 years old child, **Supplementary Table 1**) belonged to the second group [71]. The skeleton of the child was lying on its back in an extended position but the burial was disturbed by recent ploughing. The only excavated grave good was one broken bronze pin, but in other graves within the cemetery both ceramic and iron objects were excavated and these confirm the attribution of the cemetery to the Early Iron Age Mezőcsát Culture.

1.2 Radiocarbon Dating

Each of the 13 individuals reported in this work was directly radiocarbon dated at the Oxford Radiocarbon Accelerator Unit Research Laboratory for Archaeology, Oxford, UK. Details about radiocarbon dating and grave numbers of each sample are reported in **Supplementary Table 1. Supplementary Fig. 1** shows the location of the archaeological sites and radiocarbon dates in a timeline.

SUPPLEMENTARY METHODS

1 - Sampling and Genetic Analysis

1.1 Sample preparation

All bones/teeth were photographed (**Supplementary Fig. 2**) and UV-irradiated (10 minutes on each of two sides) prior to any manipulation. Their surface was then carefully removed, inside a UV cabinet, using a dremel engraving cutter attached to a

dental drill. When necessary, a dremel diamond wheel was used for subsampling. Samples were subsequently exposed to UV light for 10 minutes on each of two sides in a cross-linker (Biometra Biolink, 5 lamps at 254 nm). Fine powder was obtained by grinding the samples with a Mixer Mill (MM 400, Retsch).

All tools used in this phase were consecutively cleaned with the following products: bleach, DNA-ExitusPlus™, Ethanol and then exposed to UV for 30 minutes per side.

1.2 Petrous bones versus non-petrous bones

In order to evaluate DNA preservation in petrous bone samples versus non-petrous bones, we investigated both types of bones for seven of the 13 individuals investigated (**Fig. 1**, **Supplementary Table 2**). We compared dental crowns (IDs 8.2, 14.4, 14.7) and roots (IDs 8.3, 14.5, 14.8), ribs (IDs 8.5, 10.2), metacarpal (ID 10.6) and metatarsal portions (ID 10.4) to petrous bone parts within the same individual **(Fig. 1**, **Supplementary Table 2**). For individual NE6 we investigated two different areas of the same petrous bone (IDs 14.2, 14.3). We found that the denser part gave higher endogenous DNA yields. Therefore, this part was sampled in all other specimens. Dental portions gave the highest percentages of human DNA within the non-petrous bone samples, but the petrous bone from the same individual gave from 4- to 16-fold higher percentages of human DNA. For other non-petrous bone elements this ratio could reach up to 183-fold **(Fig. 1**, **Supplementary Table 2**).

1.3 DNA extraction

DNA extraction was carried out with around 300 mg of bone powder (**Supplementary Table 3**) and following a silica-column based protocol based on [72], as modified by [73]. Bone powder was incubated for 24 h at 55°C followed by 24 h at 37°C in 2 ml tubes with 1 ml of freshly prepared lysis buffer (final concentrations: Tris HCl pH 7.4 - 20 mM; Proteinase K, recombinant, PCR Grade – 0.65 U/ml; Sarkosyl® NL 30 – 0.7 %; EDTA pH 8 – 47.5 mM) shaking at 300 rpm in a thermo mixer (Thermomixer comfort Eppendorf®). The samples were then centrifuged for 10 min at 13,000 rpm and the supernatant was removed. Fresh lysis buffer (1 ml) was added to the pellet and the incubation and centrifugation steps were repeated. Contaminant DNA is likely to be more easily accessible during the first lysis and the second lysis buffer is more likely to include a higher relative concentration of endogenous DNA [74]. Therefore supernatant from the second lysis step was used for subsequent analyses.

The supernatant was transferred to an Amicon® Ultra-4 Centrifugal Filter Unit 30K, diluted with 3 ml of 10 mM Tris-EDTA Buffer and centrifuged for 20 min at 2,500 rpm or until a final volume of 250 µl was obtained. The flow through was discarded and 3 ml of 10 mM Tris-EDTA Buffer was added to the sample and centrifuged again for 20 – 30 min at 2,500 rpm or until a final volume of 100 µl was reached. This volume was then transferred to a silica column (MinElute PCR Purification Kit, QIAGEN) and purification was carried out following manufacturers' instructions, except for the last step in which TWEEN® 20 (at 0.05 % final concentration, Sigma-Aldrich) was added to the elution buffer (EB). Then 60 µl pre-heated EB (at 60ºC) was added to each column for the final elution. A 1 µl aliquot was used for DNA quantification with Qubit, Invitrogen, following the manufacturer's instructions (see **Supplementary Table 3**).

1.4 Library construction and amplification
Libraries were constructed from 50 µl of DNA extract, based on [75], with the following modifications. Firstly, blunt end repair was performed using NEBNext® End Repair Module (New England BioLabs Inc.), following manufacturer's instructions. Secondly, *Bst* activity was arrested through heat inactivation (20 min at 80°C).

Indexing PCRs were performed using Accuprime™ Pfx Supermix (Life Technology), with primer IS4 (0.2 µM) and an indexing primer (0.2 µM; [75]); 3 µl of library was added to the PCR mix to a total volume of 25 µl. For amplification the following temperature cycling profile was used: 5 min at 95°C, 8-12 cycles of 15 sec at 95°C, 30 sec at 60°C and 30 sec at 68°C followed by a final extension of 5 min at 68°C. PCR reactions were purified using MinElute silica columns as described above.

Quality assessment of the amplified library was performed on an Agilent 2100 Bioanalyzer, using an Agilent DNA 1000 Kit and following manufacturer's instructions. The length of the amplified fragments, excluding the adapters, ranged from 20-270 bp, with a peak around 40 bp.
Details about indices and PCR cycles used are shown in **Supplementary Table 3**.

1.5 Illumina Sequencing
All amplified libraries were first screened on an Illumina MiSeq™ platform at *TrinSeq* (Trinity Genome Sequencing Laboratory, Trinity College Dublin, Ireland), using 50 bp single-end sequencing. All libraries originating from petrous bones were subsequently sequenced on an Illumina HiSeq™ 2000 Platform at BGI (Beijing Genomics Institute, China) using 100 bp single-end sequencing. See **Supplementary Tables 2** and **3** for coverage details, number of reads and percentages of human DNA.
2 - Data Filtering, Alignment and Genotyping

2.1 Raw reads processing of Hiseq Data
Raw reads were filtered based on the indices used in library preparation, allowing a mismatch in the first base of the index sequence and a single mismatch at any other position [76,77]. The majority of template molecules were shorter than the length of the sequence reads (100 bp), so part of the P7 adapter was usually found at the 3′ ends of reads. These adapter sequences were trimmed using cutadapt v1.3 [78], allowing a minimum length of 34 bp after trimming (-m 34) and a minimum overlap of 1 bp between the adapter and the read (-O 1).

Considering the high frequency of misincorporation sites at 5' ends of the reads (C to T) in ancient DNA, as well as the complementary G to A at 3' ends [76,79,80], two bases at the 5' and 3' ends were trimmed before mapping, following [77], using the program seqtk (trimfq with options -b 2 -e 2; https://github.com/lh3/seqtk), for a final minimum length of 30bp.

2.2 Mapping
Sequence reads were aligned independently to the GRCh37 build of the human nuclear genome and the revised Cambridge reference sequence for the mitochondrial genome (NCBI accession number NC_012920.1) using Burrows-Wheeler Aligner (BWA) version 0.7.5a-r405 [81]. Apart from disabling the seed option (-l 1000), the program was run with default parameters. Duplicate reads were removed using

Samtools version 0.1.19-44428cd [82] and indels were realigned using GATK's RealignerTargetCreator and IndelRealigner [39]. Genomic depth of coverage was calculated using depth-cover (https://github.com/jalvz/depth-cover).

BAM files obtained from different sequencing lanes were merged using the MergeSamFiles Picard tool (http://picard.sourceforge.net). Duplicates were further removed and reads with a mapping quality greater than 30 were retained and used for all subsequent analyses. Alignment statistics and mean depth of coverage are presented in **Supplementary Table 3** (details on mitochondrial DNA are reported in **Supplementary Method 4**).

2.3 Preparation of modern datasets

We created a single modern SNP reference panel, labeled HGDP+, incorporating selected individuals from the Human Genome Diversity Project (HGDP) [18], a dataset used to elucidate minor the Jewish diaspora [19] and a dataset enriched with populations from the Caucasus [83].

Autosomal SNPs with rs identifiers present in all three datasets were mapped to the GRCh37 build of the human genome and genotypes were assigned to the forward strand. Datasets were merged and filtered to exclude SNPs with a minor allele frequency (MAF) $\leq 0.5\%$ and SNPs lacking a genotype call in $\geq 10\%$ of individuals. Populations relevant to the analyses were selected from this dataset (**Supplementary Table 4**) and outliers were identified using EIGENSOFT's smartpca program (pairwise $r^2 > 0.2$ [84]) and removed. The final dataset contained 517,623 autosomal SNPs genotyped in 552 individuals.

2.4 SNP calling in ancient samples

To explore the ancient data in the context of modern variation we called genotypes at all positions that overlapped with the HGDP+ dataset. Different SNP calling procedures were followed for the high coverage and low coverage data.

For the high coverage genomes NE1 and BR2, diploid genotypes were called for all sites in the HGDP+ dataset using GATK's UnifiedGenotyper tool [39]. Genotypes were only called for alleles observed in the HGDP+ dataset and using only sequence data with a base quality ≥ 20 and depth ≥ 15. To exclude false genotype calls due to molecular damage, a minimum allele count of 4 was required for the T or A alleles of C/T or G/A SNPs, respectively. Filtered VCF files were transformed to PED format using VCFtools [85] and merged with the modern dataset using PLINK, retaining only SNPs present in the ancient sample (**Supplementary Table 5**). For principal component and admixture analyses one allele was randomly chosen at each called locus, creating homozygous genotype calls.

For the low coverage genomes, genotypes were obtained for principal components analysis (PCA) following [3,86] using the GATK [39] tool Pileup. The resulting pileup files were filtered for a minimum base quality of 15. Triallelic genotypes and genotypes that could be explained by molecular damage were removed (A where the alternative state is G and T where the alternative state is C). For sites with > 1× coverage, one base call at that site was randomly selected. Each hemizygous allele call was duplicated to form a homozygous diploid genotype and PED format files were created which were merged with the modern datasets as above. For each SNP genotyped in

the ancient sample, one allele was randomly selected from each individual’s diploid genotype in the modern dataset, simulating hemizygous data. This allele was then duplicated to generate a homozygous diploid genotype.

The number of individuals and SNPs present in each merged modern/ancient dataset is reported in **Supplementary Table 5**. These SNPs were used in downstream analysis such as PCA for all samples and runs of homozygosity for the high coverage genomes (**Supplementary Method 6** and **5**, respectively).

3 - Authenticity of Results

3.1 Independent replication

Results obtained in Dublin were partially replicated at the Department of Biology, University of York for two portions of the temporal bones from individuals NE1 and BR2, using around 250 mg each. DNA was extracted following [87]. Libraries were built following [75], with the following modifications: (a) the P5 adapter included an index sequence of 8 nucleotides at the 3’ end (NE1 - ACCAACCG, BR2 - AACGCATC), and (b) the filtration step between the blunt end repair and the adapter ligation was substituted by heat inactivation of the enzymes. Libraries were amplified in two rounds using AmpliTaq Gold DNA Polymerase. Eight parallel amplifications of 15 cycles each were carried out using the primers IS7-indPCR and IS8 [75]. Subsequently, 2.5 µl of the library were used as template for a second amplification of 5 cycles with primers IS4 and P7-indexing (NE1 - ACCAACCG, BR2 - AACGCATC, the same indexes used for the P5 adapter).

These libraries were subsequently sequenced on a MiSeq platform and reads were processed following the procedures described in **Supplementary Method 2**. We obtained 679,400 (18.49%) and 1,027,012 (26.96%) reads aligned (duplicates removed) to the human autosomal genome for NE1 and BR2 respectively.

GATK’s UnifiedGenotyper [39] was used to call HGDP+ variants from quality trimmed bam files, restricting the panel of variants to those called in the high coverage genomes (**Supplementary Method 3**). As a measure of result reproducibility, SNPs called in the replication analysis were then compared to corresponding SNPs in the high coverage genomes. Due to the low coverage data obtained in the replication, only homozygous calls in the high coverage genomes were considered, resulting in a comparison of 2,249 genotypes for NE1 and 3,609 genotypes for BR2. Our results were highly congruent. A concordance rate of 99.69% was found for NE1 and a similarly high rate of 99.08% was recorded for BR2. Only one and four SNPs in the low coverage data could not be potentially explained by molecular damage for NE1 and BR2 respectively (0.04% and 0.11%) and might therefore be due to contamination or genotyping error. This test supports high reproducibility of our results (over 99%) and an extremely low laboratory contamination rate (below 0.11%).

Mitochondrial DNA reads were also separately aligned to the rCRS as described in **Supplementary Method 2**, giving mean coverages of 0.95x for NE1 and 1.42x for

BR2. The same haplogroup determined using the high coverage data was also predicted for these low coverage replicated samples, albeit with a lower associated probability (NE1, U5b2c, probability 51.1%; BR2, K1a1a, probability 61.6%).

3.2 Negative controls analysis

During the screening MiSeq run (**Supplementary Method 2.5**), contamination controls were sequenced in parallel with the samples. One extraction control, one library control and two PCR negative controls were prepared for each seven samples. Moreover, air controls (1.5 ml / 2 ml tubes were opened in the drilling working area for 30 minutes, prior to initiation of sample preparation, to check the quality of the air) and water controls (1 ml of water was added to clean grinding jars and shaken, to check they were contamination free) were periodically prepared as well.

To estimate the laboratory contamination at each stage, we compared the number of reads alignable to the human genome obtained for the controls with the number of reads obtained for the samples analyzed here, normalized by the total number of reads obtained in each MiSeq run. The contamination fraction (C) was calculated as following:

$$C = (R_b / TV_b) / (R_s / TV_s) \qquad (1)$$

where R_b denotes the number of control reads aligning to the human genome, R_s the number of reads aligned to the human genome for the sample analyzed in parallel with the control, T the total number of reads obtained in the specific Miseq run and V the volume added to the pool loaded on the sequencer for controls (V_b) and samples (V_s). Results are reported in **Supplementary Table 6**. For each sample**,** detailed information about relevant controls is provided.

3.3 Estimation of molecular damage and sequence length

Sequence length and molecular damage were calculated by considering a subsample of 500,000 reads per sample. Adapter sequences were trimmed and reads with a minimum length of 30 bp were retained (**2.4**). These sequences were mapped to the human genome as described in **Supplementary Method 2.2**; however no mapping quality filter was applied. The resulting bam file was used as input for mapDamage 2.0 [41,88] with a minimum base quality of 20 (option -Q20, Phred score) specified.

The mean and standard deviation of sequence lengths are reported in **Supplementary Table 7**, while the C to T patterns at 5’ end are reported in **Supplementary Fig. 3**. Both results point to authenticity of ancient molecules, with an average length of 50-70 bp, typical for ancient DNA [89], and an upsurge of C to T transitions at 5’ ends. A reciprocal pattern of increased G to A transitions is observed at 3’ ends. The values for 5’ C to T transitions lie between 0.147 and 0.284, typical for samples > 500 years and up to 50,000 years old [90].

3.4 Contamination estimates on the mtDNA chromosome

Mitochondrial DNA contamination was estimated using high quality reads that aligned to the rCRS sequence (**Supplementary Method 2.2**). Only bases with a quality greater or equal to 23, mapping quality above 30 and sequence length higher than 30 were analyzed. Bases that matched the consensus base call (**Supplementary Method 4.2**) at a position were considered primary bases. Bases that diverged from

the consensus base were considered secondary bases. The contamination rate (as percentage of contamination, % C, **Supplementary Table 8**) was calculated as the average of the number of secondary bases at haplotype defining positions that could not be explained by molecular damage (C to T or G to A, >82.35% of the total number of mismatches), following [86]. Given the high transition / transversion ratio of mtDNA, also the estimates based on all sites (including C to T and G to A possible due to damage) are provided (as percentage of contamination including possible molecular damage, % C+MD, **Supplementary Table 8**).

The contamination fraction (C) was calculated as following:

$$C = (D_i/D_t) / P_t \qquad (2)$$

where D_i is the depth of secondary base at a haplotype defining site where the secondary base cannot be attributed to damage; D_t the total depth of coverage at that site and P_t the total number of haplotype defining positions evaluated.

Our contamination estimate, which might include sequencing errors as well as possible heteroplasmy and NUMTs (mtDNA transpositions into the nuclear DNA), span from 0 to 0.81%, suggesting a very low mtDNA contamination rate. See **Supplementary Table 8** for details.

3.5 Contamination estimates on the X chromosome

For the six male individuals studied here it was possible to estimate X chromosome contamination. The X chromosome is a haploid marker in males and thus any polymorphic sites identified may be due to contamination, DNA damage, sequencing errors or mis-mapping. Contamination is expected to be more transparent at polymorphic sites than at monomorphic sites as there is greater opportunity for variability in contaminating population(s). In contrast, sequencing error should affect all positions of the genome in the same way.

In order to assess the contamination rate we examined discordance in the rate of heterozygous calls between known polymorphic sites and their adjacent sites. We used a set of polymorphic markers, reported in the 1000 Genomes panel [28], on the non-pseudoautosomal region of the X chromosome. The frequency of the minor allele in Europe was used to isolate sites, which exhibited polymorphism in European populations. As the samples originate from Hungary and have been mainly handled by European individuals, we investigated the probability of contamination from a European source.

The aforementioned 1000 Genomes dataset (957,967 SNPs) was filtered as follows:

1. Polymorphic sites were required to be at least 10bp apart
2. Observed polymorphism was required in European populations, i.e. minor allele frequency > 0
3. Insertions and deletions were removed

This resulted in a panel of 378,470 SNP positions for subsequent analyses. For each of the ancient male samples, GATK's pileup [39] was used to call these positions in quality trimmed bam files. Reads with high mapping quality (Q30) were used to

negate the effects of mis-mapping. The resulting PILEUP file was filtered as outlined below:

1. Only bases with quality ≥ 20 were considered
2. Sites were required to have a minimum depth of coverage of 5 (for the ~20× sample BR2) or 3 (for the ~1× samples KO1, NE5, NE6, NE7, IR1) and a maximum depth of 40
3. Tri-allelic and tetra-allelic sites were removed
4. Sites in which the reference allele frequency was equal to the alternate allele frequency were discarded

We performed Test 1 and Test 2 as described in [91]. For Test 1 we computed the number of primary (most common) and secondary (least common) alleles present at each SNP site as well as for the four sites upstream and downstream of this site (**Supplementary Table 9**) as a proxy for background error. A contingency table (**Supplementary Table 10**) was prepared for each sample.

Using Test 2 we remove the assumption of independent error rates at each site by randomly sampling a single read at each site. We computed again the number of primary (most common) and secondary (least common) alleles as well as the four sites upstream and downstream (**Supplementary Table 9**) and prepared a contingency table (**Supplementary Table 10**) for each sample.

3.5.1 Maximum Likelihood Contamination estimate on the X chromosome

We took the average observed probability of error (e) at adjacent sites as the background error rate. The probability of observing a secondary allele (P_s) at this SNP site is given by:

$$cf + (1-c)e \qquad (3)$$

where c is the contamination rate and f is the frequency of the secondary allele in Europe.

Assuming that the number of secondary alleles follows a binomial distribution, the likelihood of observing the distribution of our data is proportional to the probability of observing a secondary allele and the probability of observing a primary allele at each site:

$$(cf + (1-c)e)^s\,(1-(cf + (1-c)e))^p \qquad (4)$$

where s and p are the secondary and primary allele count at each site. The likelihood to observe the distribution of our data is given by the product of all these probabilities at each site:

$$\Pi\,(cf + (1-c)e)^s\,(1-(cf + (1-c)e))^p \qquad (5)$$

We transformed this product to the sum of its logarithm and by examining the maximum likelihood surface determined the contamination rate to be between 0.21 - 1.20% (**Supplementary Table 11**).

3.6 Conclusions

Authenticity of the sequences obtained is of key importance in any ancient DNA study. However, the probability of contamination differs with the types of samples investigated, with ancient human samples being particularly prone to contamination [92]. Despite this, and the fact that early NGS studies on ancient human remains revealed problems of contamination [93], many projects rely solely on *a posteriori* in-silico assessment of contamination levels. We have applied both *a priori* (extraction and library construction controls) and *a posteriori* (bioinformatics assessment of contamination levels) controls. We also performed classical replication of results in a secondary laboratory, a widely accepted measure to control for contamination in pre-NGS studies, which has thus far not been applied to NGS studies. All our analyses reveal consistent and extremely low levels of contamination of ~ 1%, or often much less.

4 - Sex determination and uniparental ancestry

4.1 Molecular sex determination

Sex was determined by analyzing the ratio of X to Y chromosome reads following [42]. All samples could be clearly assigned to one of the two sexes (**Supplementary Fig. 4**).

4.2 Mitochondrial DNA

All samples were mapped to the mitochondrial DNA (mtDNA) genome and filtered as described in **Supplementary Method 4.2**. BAM files were analyzed with the online tool MitoBamAnnotator [94], using default parameters (minimal base quality of 23, minimal base quality per read of 5, maximal mismatch fraction of 3/36, maximal indel fraction of 3/36, maximal number of N's in a read of 0) except for the minimal mapping quality (30) and minimal read length (30 bp), already filtered in the input BAM file (**Supplementary Method 3.2**).

The minimum depth of coverage for establishing the consensus haplotype was 3, after filtering for molecular damage (**Supplementary Table 12**). Corresponding haplogroups were obtained through the online tool Haplogrep [95] based on the build 15 phylogeny of PhyloTree [96].

KO1 shows the rare maternal lineage R3, which has only been described in one Finn [97], one Armenian (Family Tree database, http://www.familytreedna.com/) two Yakuts and one Bengali individual[98]. New data support the placement of this haplogroup within the R1 branch and it has been recently suggested to rename this haplogroup as R1b [98]. Little is known about this haplogroup from modern populations. The finding of an Early Neolithic specimen from this branch suggests a molecular dating closer to the upper boundary of the range proposed by [97], 12,245.8+-6,768.4 BP.

KO2 belongs to haplogroup K, the most frequent haplogroup found in the time series, together with NE6, BR1 and BR2. This haplogroup is present in around 6% of Europeans and Near Eastern populations (88), reaching higher frequencies in the Druze (16%) [99,100] and the Ashkenazi Jewish (32%) populations [101]. It has been already described in ancient European Neolithic specimens [23,24,102–106] and the Late Neolithic/Copper Age specimen from the Alps, Ötzi, belongs to the same sub-clade K1 [6]. The KO2 specific haplogroup is K1 and the sub-clades K1a and K1c were found also in later samples from the Hungarian timeseries: the Neolithic specimen **NE6** (K1a3a3) and the Bronze Age **BR1** (K1c1) and **BR2** (K1a1a). This might suggest a certain maternal continuity through time on the Great Hungarian Plain.

Individual **NE1**, dated to the Middle Neolithic ALP culture, belongs to the U5b2c mtDNA haplogroup. The clade U5 has been associated with the Palaeolithic background of Europeans and is currently widely distributed in Eurasia, showing a frequency of around 9% in Europeans [99]. It shows frequency peaks in the Basque country [107] and in Scandinavia, with the sub-clade U5b being present in 41% of the Saami population [108]. It has been suggested that this haplogroup might have spread into Europe after the Last Glacial Maximum from Palaeolithic refugia [109]. The presence, at low frequencies, of this clade outside Europe, in northern Africa and the Near East is probably due to back-migration from the European continent [99,108]. These hypotheses emerging from modern population genetics seem to be supported by ancient DNA results: haplogroup U5 assignments have been described in all published European pools of Mesolithic specimens [5,8,86,110,111]. However, it has also been described in Neolithic populations at lower frequencies [102,103,105,106]. These data, together with our detection of this haplogroup in the Middle Neolithic Hungarian specimen NE1, might suggest population admixture at the beginning of the Neolithic.

NE2 together with **CO1** show haplogroup H, the most common clade among current Europeans (40-60%), with decreasing frequencies towards the Caucasus and the Near East (88). However, it has not been observed in Mesolithic hunter-gatherers and shows a lower frequency among the first Neolithic farmers (~19%) [112]. It has been suggested that its frequency started to increase during the Middle and Late Neolithic [112] and this is consistent with our observations here.

The sample **NE3** belongs to haplogroup X2, which is a widespread haplogroup around the globe. The majority of X-clade individuals in Europe, the Caucasus and the Near East belongs to the sub-lineage X2 [113]. Modern population genetics suggest an association of this clade with a Palaeolithic background [113], but it has been described only in post-Neolithic samples [106,114,115].

NE4 and **NE5** belong to haplogroup J (J1c and J1c1 respectively), a widespread haplogroup in Eurasia showing a frequency cline from the Near East (13%) towards Europe (9%) [99,116]. Sub-haplogroups J1b and J1d are generally Near Eastern, while J1c is mainly European, specifically from central Europe, the Balkans and Ukraine, where it represents 80% of J1 [116]. Haplogroup J (in some cases sub-typed as J1 or J1c) was found in other Neolithic European specimens [23,24,104,106,110,117] as well as from one Northern European Neolithic hunter-gatherer [111]. Clade J1c probably developed within Europe and spread during the Neolithic.

The Late Neolithic specimen **NE7** belongs to haplogroup N1a, a very rare haplogroup in Europe (0.2%) [118]. However, this haplogroup had a very high frequency in Early Neolithic group farmers from central Europe [24]. Specifically, it was found in 5/22 German LBK samples as well as a Hungarian AVK individual from Ecsegfalva I [24]. The NE7 haplotype belongs to the so-called European branch, as well as the German LBK samples, while the published Hungarian ancient sample showed the Central Asian sub-branch [118]. The authors of [24] suggested that this was the result of a Neolithic diffusion by pioneer farming groups. Considering that it has never been typed in pre-Neolithic specimens, it seems that this haplogroup had a strong association with the Linear pottery cultures, both LBK and AVK.

The youngest sample of our time series, **IR1,** belongs to haplogroup G, specifically the sub-clade G2a1. Haplogroup G is typically East Asian [119]. G2 is found in northern China and central Asia, reaching peak frequencies in South Siberian populations [119]. This points clearly to an Asian maternal ancestry of the specimen IR1. The archaeological context supports this hypothesis; IR1 has been associated with the pre-Scythians, a culture linked to immigration from the East [120].

4.3 Y-chromosome

We determined Y-chromosome haplogroups by checking the SNP set of the ISOGG (International Society of Genetic Genealogy Y-DNA Haplogroup Tree 2014, Version: 9.22 Date: 13 February 2014 http://www.isogg.org/tree/) in all male samples namely KO1, NE5, NE6, NE7, BR1 and IR1.

The dataset was filtered by removing all SNPs without position information, those marked as under investigation and to mitigate strand issues - all CG and TA SNPs. This resulted in a final set of 663 SNP positions. In **Supplementary Table 13** we report haplogroup assignments using between 6 and 12 informative positions per individual.

KO1 was assigned to haplogroup I2a. Y-chromosome clade I is widespread in Europe with frequency peaks of 40%–50% in Scandinavia and southern Europe [121]. We can see parallels with his mtDNA assignment, U5, as the Y-chromosome I haplogroup is also virtually absent outside Europe [122]. Modern population genetics suggests a European Upper Palaeolithic origin of haplogroup I [121], which is currently supported by ancient DNA, as five Mesolithic specimens from central and northern Europe have been typed as belonging to the I lineage [5]. Our data are consistent with the above and suggest that KO1 had a Mesolithic paternal ancestry.

Interestingly, haplogroup I reaches very high frequency in Sardinians (40.7%) [123], specifically the I2a1a sub-haplogroup, which is virtually absent elsewhere [123]. Haplogroup I was probably introduced there during the Neolithic by farmers with a Mesolithic paternal ancestry, and possibly reached high frequency due to genetic drift. **NE7** also belongs to haplogroup I2a. However, it is interesting to note the contrast in maternal and paternal ancestry of this specimen, which might suggest patrilocality practices. NE7 showed a Y-chromosome lineage associated with the Palaeolithic background and a mtDNA clade (N1a1a1a) associated with first Neolithic groups in central Europe [24].

Both **NE5** and **NE6** belong to the same haplogroup C6 (also called C1a2 or C7). Haplogroup C has been described mainly in southern and eastern Asia, but some branches have also been found in Oceania, Malaysia, the Americas and Europe. The distribution of this clade has been suggested to support an African origin followed by a single migration 'Out-of-Africa' firstly towards India and then to southeast Asia and the other continents [124]. Only recently a new European branch has been identified, C6 (initially called C7), in southern Europe [125]. Further investigation will be needed to determine if it represents an ancient European lineage or it constitutes a recent Asian signal [125]. The first hypothesis is supported by the identification of this subclade in an ancient Mesolithic specimen [7] from northern Spain. These data suggest: (a) this currently uncommon and not well known haplogroup was probably much more common in the past, (b) it was widespread in Europe, as it has been found in eastern and Western European ancient specimens and (c) its distribution and frequencies have been significantly changing through time.

BR2 was classified as J2a1. Haplogroup J is currently frequent in the Fertile Crescent and the Caucasus, showing decreasing frequency patterns to southern Europe and Iran [121]. Because of these patterns it has been traditionally associated with the Neolithic diffusion from the Near East [126,127], but this simplistic interpretation does not recognize the complexity of both geographic patterns and phylogenetic resolution [128]. Post-Neolithic migration has been suggested to be responsible of the spread of sub-haplogroup J2a1b1 [129,130], probably during the Bronze age, together with the spread of the Maritime Troy culture [131]. This last hypothesis might explain the detection of this haplogroup only during the Late Bronze Age.

IR1 belongs to clade N, which is typically northern Eurasian and virtually absent elsewhere [132,133]. It has been suggested that this haplogroup originated 12,000-14,000 years ago in southeast Asia followed by a subsequent spread toward northeastern Europe and Siberia [133]. It is widespread in Siberia but absent in Native Americans, dating the spread of this haplogroup after the Bering strait crossing towards the Americas [133]. These data might suggest an Eastern paternal lineage of IR1, in agreement with the archaeological link with Steppe groups and also an Asian maternal ancestry based on its mitochondrial haplogroup G2a1.

5 - Imputation of ancient genomes

Imputation is a method commonly used in genomics to infer missing genotypes based on comparison of their surrounding haplotypes with those of a larger dataset (often a phased reference panel). Imputation software can typically take genotype probabilities or likelihoods as input data, and it has been shown recently by Pasaniuc *et al.* [27] that even low-density variant likelihood calls made with ultra low-throughput sequence data are sufficient for an imputation engine such as Beagle [40] to make accurate estimates of both missing and low coverage genotypes. Output data represent the probabilities of the possible genotypes; suitable thresholds can therefore be chosen to control the error rate in the resulting data.

Imputation may be an effective tool for the analysis of low-coverage ancient genomes, as a dense set of accurate diploid calls may be made using the available low-coverage sequence data.

5.1 Assessment of imputation using subsampled data

Imputation was evaluated using high-coverage sequence data from individuals NE1 and BR2, subsampled using SAMtools [82] to depths of approximately 0.1×, 0.25×, 0.5×, 0.75×, 1× and 2.5×.

Imputation was initially assessed using data from chromosome 22. A phased SNP call set from the phase 1 integrated release of the 1000 Genomes Project was used as a reference panel for imputation. Genotypes were imputed by Beagle [40], using default parameters and intervals of 1 Mb. The resulting genotype probabilities were converted to PLINK-format BED genotype data for a range of imputed genotype probability thresholds (0.33 to 1). Any SNP without a genotype exceeding the threshold had a missing genotype assigned for that individual. Imputed genotypes on chromosome 22 were compared to confident high-coverage genotype calls made at well-characterized SNP sites called in the HGDP+ SNP sites (**Supplementary Method 2**) in samples NE1 and BR2 (**Supplementary Method 2**). **Supplementary Fig. 5 a** and **c** show the percentage of imputed genotypes matching high-coverage calls, broken down by genotype. The homozygous reference state was the most accurately imputed genotype, followed by homozygous alternate, then heterozygotes. Increasing the imputed genotype probability threshold resulted in increased accuracy, but at the cost of retaining fewer genotypes (**Supplementary Fig. 5 b** and **d**). Consistent patterns were observed for both high-coverage genomes.

Taking NE1 1× data as an exemplar, imputed data were marginally more accurate if the SNP site had spanning sequence data (**Supplementary Fig. 5 e**). When percentage of genotypes meeting threshold was plotted against coverage for two representative imputed genotype probability thresholds (**Supplementary Fig. 5 f**), a plateau was observed in the increase of retained genotypes commencing around 1×. This corroborated our target depth of 1× for several of the samples sequenced; extra sequencing would yield a diminishingly small increase in data.

To assess whether bias is introduced during imputation, genome-wide imputed genotypes (genotype probability threshold 0.99) from subsampled NE1 and BR2 datasets were merged with the HGDP+ modern reference dataset (**Supplementary Method 2**) and assessed using principal components analysis (PCA, **Supplementary Method 6**) and runs of homozygosity (ROH, **Supplementary Method 7**).
PCA plots created using the subsampled datasets and high coverage genotypes from the same sample were merged using Procrustes transformation (**Supplementary Method 6**). Imputed data fall within a range of genome-wide variation defined by principal components 1 and 2 that is virtually indistinguishable from that of their corresponding high-coverage genotypes (**Supplementary Fig. 6**). The total genome length under ROH using imputed and high coverage data were compared to the distribution of this statistic for the HGDP+ panel measured using the same loci. The percentile position of the ancient samples was found to be reasonably robust to the process of imputation from low coverage sequence. The range in each was within 2.7% (**Supplementary Table 14**, **Supplementary Fig. 7**)

5.2 Imputation of low-coverage genomes

For genomes sequenced at 1× and 0.1×, genotype likelihoods were called using GATK's UnifiedGenotyper [39] for alleles observed in the 1000 Genomes Project at the 28,627,866 autosomal SNP sites used for the subsampled data. Any SNP with a genotype potentially de
rived from a deaminated cytosine was reset to have equal likelihoods (0.3333) for all three genotypes. SNPs with no spanning sequence data also had all three genotype likelihoods set at 0.3333. Genome-wide variants were imputed in intervals of 1 Mb by Beagle version 3.3.2 [40] using a phased SNP call set from the phase 1 integrated release of the 1000 Genomes Project as a reference panel [28]. For genome-wide analyses such as PCA (**Supplementary Method 6**) and ADMIXTURE analysis (**Supplementary Method 8**), genotype probabilities were converted to PLINK-format BED data, imposing a genotype probability threshold of 0.99. For single-locus analysis of selective sweeps (Supplementary Method 9), a genotype probability threshold of 0.85 was used to determine the genotype.

5.3 Assessment of Ancient Samples in the Context of the 1000 Genomes Data

As imputation relies on the presence of representative haplotypes in the reference panel, a potential weakness of this approach could be that haplotypes found in ancient samples may represent extinct lineages that are absent in modern populations, resulting in decreased accuracy of imputation for divergent samples. As KO1 and IR1 appear as outliers when examined in the context of the HGDP+ dataset (**Fig. 2**) we analysed these samples, as well as NE1 and BR2, in the context of the 1000 Genomes data using PCA (**Supplementary Fig. 8**) We found that KO1, NE1 and IR1 all fall outside clusters of the 1000 Genomes individuals, suggesting that this dataset is as representative for KO1 and IR1 as it is for NE1. As ancient high coverage data becomes more accessible it will be possible to directly assess imputation accuracy for diverse samples.

6 - Principal Components Analysis

To examine the samples in the context of the variation found in the HGDP+ dataset we performed individual principal component analysis (PCA) on all 13 samples (**Supplementary Method 2.3**) using SMARTPCA software [3,84]. Individual PCAs were plotted with R [134] with PC1 and PC2 vectors transformed to match the configuration of the HGDP+ dataset (data not shown).

6.1 Procrustes transformation

In order to visualize non-overlapping data from all samples jointly without compromising the resolution of the picture, we used an approach based on Procrustes analysis [3] using the R package 'vegan' [135]. We plotted the average of the transformed coordinates for individuals in the HGDP+ dataset with each of the ancient samples transformed coordinates. The final plot, **Fig. 2** in the manuscript, was obtained with R [134] and modified with Inkscape version 0.48.3.1 (http://www.inkscape.org/). Population and continent codes used in the PCA plots are shown in **Supplementary Table 4**.

6.2 PCA with imputed data

The SNPs typed in the HGDP+ dataset (**Supplementary Method 2.3**) were selected from imputed genome-wide data, including the two high coverage genomes (samples NE1 and BR2) subsampled to 1× and the seven ~1× coverage genomes (samples KO1, NE5, NE6, NE7, CO1, BR1 and IR1). To get an accurate call of heterozygotes (**Supplementary Method 8**) the imputed genomes were filtered by a probability cutoff of 99%. PLINK [136] was used to merge the ancient and modern data without allowing missingness, giving a total of 151,407 shared SNPs across all samples. The PCA plot obtained (**Supplementary Fig. 9**) closely reflects the original PCA presented in **Fig. 2**, allowing cross validation of the alternative approaches.

7 - Runs of Homozygosity

Runs of homozygosity (ROH) are extended homozygous haplotypes that are identical by descent, arising due to consanguinity, restricted population size and selection events [137]. The portion of the genome under long ROH increases with inbreeding. A predominance of shorter ROH is indicative of long-term reduced population size [138]. Pemberton *et al.* [30] detected superimposed distributions of ROH length in human populations, with a cut-off of ~1.6 Mb distinguishing longer and shorter runs.

7.1 Length of ROH analysis

The ROH analysis was carried out with PLINK [136] with the following specifications:

- A sliding window of 5000 kb (--homozyg-window-kb 5000)
- A sliding window of 50 SNPs (--homozyg-window-snp 50)
- One heterozygote allowed in a window (--homozyg-window-het 1)
- A proportion of 0.05 of overlapping windows that must be called homozygous to define any given SNP as 'in a homozygous segment' (--homozyg-window-threshold 0.05)
- A minimum number of 50 SNPs to be called as homozygous (--homozyg-snp 50)
- A minimum number of 500 Kb to be called as homozygous (--homozyg-kb 500)
- If two SNPs within a segment were > 100 kb apart, that segment was split in two (--homozyg-gap 100)
- A required minimum density of 50 SNPs (--homozyg-density 50)

Short (< 1.6 Mb) versus long (≥ 1.6 Mb) ROH were plotted in **Fig. 5 b**.

The extreme position of KO1 in **Fig. 5 b** suggests a small population size and higher degree of shared ancestry in its population of origin. The remaining samples are progressively closer to modern population variation, with the most recent samples dating to the Iron, Bronze and Copper Age (IR1, BR2, CO1) giving values typical of modern individuals.

Supplementary Table 14 shows the comparison between high coverage and imputed data for NE1 and BR2. When the total genome length under ROH in each is compared to the distribution of this statistic for the HGDP+ panel measured using the same loci, the percentile position of the ancient samples was found to be reasonably robust to the process of imputation from low coverage sequence. The range in each was within 2.7%.

7.2 Correlation between ROH and Age

In **Fig. 5 b** samples seem to be distributed according to their age. We investigated the correlation between sample age and the total length of ROH and we estimated the coefficient of correlation r^2 (0.4042) and the p-value (0.06573) fitting the points into a linear model (**Fig. 5 a**). Even though the p-value is borderline significant, we can see a trend for decreasing total Mb of ROH through time. These results probably reflect an increase in mobility, population size and outbreeding over time [139].

8 – Admixture analysis

8.1 ADMIXTURE software

The unsupervised clustering algorithm implemented in ADMIXTURE [29] was used to estimate the ancestral genetic components of the seven ~1× imputed samples plus the two 1× sub-sampled ~20× genomes along with 552 modern reference samples (**Supplementary Method 7.1**). Prior to the ADMIXTURE analysis, the SNP dataset was filtered in PLINK [136] to remove SNPs in pairwise LD using a r^2 value of 0.2. This analysis resulted in a final dataset of 60,824 SNPs. An exploratory ADMIXTURE analysis was first completed using 10 replicates for each value of K({1..10}), this analysis returned K=4 to be the number of clusters that provided the optimal minimisation of the CV error. The K=4 analysis was then repeated 100 times and the results analyzed in CLUMPP using the *Greedy* algorithm with 1,000 random permutations [3]. The 100 runs produced similar results with a maximum similarity coefficient H’ of 0.9933177. The clustering results were then visualized using R [134] (**Fig. 4**).

8.2 NgsAdmix software

NgsAdmix allows for the production of admixture analyses from low coverage genomes [140], instead genotype likelihoods are used. To assess the impact of imputation on the ADMIXTURE analysis, the NgsAdmix analysis was completed on the same samples.

Prior to the NgsAdmix analysis, the ~1× bam files used for the imputation were converted to genotypes likelihoods using ANGSD (http://www.popgen.dk/angsd/index.php/Main_Page), specifically to genotype

likelihood beagle input file format. These files were then merged with the HGDP+ dataset in the same genotype likelihood format and NgsAdmix run with default settings [140]. The clustering results (**Supplementary Fig. 10**) reflect the ones obtained using ADMIXTURE (**Fig. 4**).

9 - Analysis of selective sweeps

9.1 Pigmentation

Skin reflectance increases with distance from the equator [141] suggesting a selective response to restricted environmental ultraviolet radiation, required to synthesize vitamin D. Several genes involved in pigmentation have been investigated [142–144] and the available results suggest a complex genetic history of these genes, with different selective pressure in space and time [32,145–148].

Here we report the imputed genotype of three pigmentation genes (SLC24A5, SLC45A2, associated with skin color, and TYRP1, associated with iris and hair pigmentation; **Fig. 3**) in which three SNPs (rs1426654, rs16891982 and rs2733831 respectively) have been specifically selected in Europeans. The selected alleles of the former two are close to fixation in Europe. The time depths for selective sweeps of these three SNPs have been proposed as 11,000-19,000 years ago based on modern population data [33], while recently it has been suggested that positive selection on these variants were still ongoing later, based on ancient samples from the Pontic–Caspian steppe dating 6,500–4,000 years ago [32].

Within the Hungarian time series, we observe the appearance of the SLC45A2 selected allele in a homozygous state during the Middle Neolithic period, whereas the selected allele in the gene SLC45A2 appears even later, towards the end of the Neolithic (**Fig. 3**). This suggests in both cases that the selective sweep occurred later than previously hypothesized [33], as suggested by [32]. The selective sweep at the gene TYRP1 is more difficult to identify, given that the current frequency of the selected allele is not close to fixation. However its frequency in Neolithic samples is 0.375 (6/16 alleles, **Fig. 3**) while in subsequent periods it doubles to 0.75 (6/8 alleles, **Fig. 3**), suggesting positive selection on this allele.

9.2 Lactase persistence

In mammals the ability to digest raw milk usually declines after weaning, but several human populations are lactase (LCT) persistent, with an ability to digest milk during adulthood [149]. Their current geographical distribution is not uniform and correlates with historical milk consumption [150]. In Europeans, a selective sweep is strongly associated with the T allele at SNP rs4988235 [151,152], which regulates LCT expression persistence. Its presence in either homozygous or heterozygous form allows milk digestion into adulthood. It has been suggested that the T allele was selected during the Neolithic, around 7,500 years ago in a region between the central Balkans and central Europe, possibly within the Neolithic LBK culture in central Europe [150]. However, this allele has so far not been detected in Neolithic LBK samples [5,153], nor in Late Neolithic samples from northeastern Iberia [38] and only in 5% of the later Pitted-Ware Culture hunter-gatherers from Sweden [154]. Our samples confirm the

absence of the selected allele in the Neolithic (**Fig. 3**). The T allele is first detected in the Late Bronze Age, just over 3000 years ago.

10 - Phenotypic Traits

We have implemented two prediction models developed in forensic science for our pool of ancient genomes: the 8-plex [155,156] and the Hirisplex [157] systems. We used the former to infer the pigmentation of skin and the latter for eye and hair color prediction, based on 24 variants. We could infer phenotypic traits of all individuals studied (**Fig. 3**). The genotyping was performed on observed data for the two high coverage genomes, while imputed genotypes were used for the 11 low coverage genomes, after checking their compatibility with observed data.

10.1 The 8-plex system

Results for the 8-plex system are reported in **Supplementary Table 15**. The analysis of skin pigmentation was inconclusive for seven of thirteen samples, but it is estimated that samples NE4, NE6, NE7, CO1, BR2 and IR1, all from latter phases of the timeline, had light skin. This multiplex also allows eye color inference, but these results are not reported as they provide a lower level of accuracy than the Hirisplex system (Supplementary Method **10.2**).

10.2 The Hirisplex system

Probabilities associated with each phenotype using the Hirisplex system are shown in **Supplementary Table 16** and details about the observed and imputed genotypes are reported in **Supplementary Table 17** and **Fig. 3**. Eye color prediction could be obtained for all samples and KO1 and NE6 are predicted as displaying blue eyes and NE7 probably showed an intermediate phenotype. The other ten samples most likely had brown eye color.

Hair color has been predicted to be black, dark brown or light brown for the majority of the samples (11 out of 13, **Fig. 3**). The blue-eyed Neolithic specimen, NE7, probably had blonde hair, as did the brown-eyed Iron Age sample, IR1.

10.3 Conclusions

These results suggest that light skin was present in Europe during the Neolithic. Moreover, we can distinguish a loose trend in hair lightening through time, whereas an opposite trend is detected for eye color. The most ancient sample KO1 was predicted to have a blue eye phenotype, while all the other Early and Middle Neolithic specimens were assigned as having brown eyes. It is only in the late Neolithic (samples NE6 and NE7 respectively) when intermediate and blue eye colors reappear in our transect. Considering the genetic Mesolithic ancestry of KO1, this pattern concurs with a higher frequency of this phenotype in pre-Neolithic times. To date only two Mesolithic samples have been typed for this trait, one from central Europe [5] and one from southeastern Europe [7], both predicted to have blue eyes.

SUPPLEMENTARY REFERENCES

1. Sánchez-Quinto, F. *et al.* Genomic Affinities of Two 7,000-Year-Old Iberian Hunter-Gatherers. *Curr. Biol. CB* (2012). doi:10.1016/j.cub.2012.06.005
2. García-Garcerà, M. *et al.* Fragmentation of Contaminant and Endogenous DNA in Ancient Samples Determined by Shotgun Sequencing; Prospects for Human Palaeogenomics. *PLoS ONE* **6,** e24161 (2011).
3. Skoglund, P. *et al.* Origins and Genetic Legacy of Neolithic Farmers and Hunter-Gatherers in Europe. *Science* **336,** 466–469 (2012).
4. Raghavan, M. *et al.* Upper Palaeolithic Siberian genome reveals dual ancestry of Native Americans. *Nature* **505,** 87–91 (2013).
5. Lazaridis, I. *et al.* Ancient human genomes suggest three ancestral populations for present-day Europeans. (2013). doi:http://dx.doi.org/10.1101/001552
6. Keller, A. *et al.* New insights into the Tyrolean Iceman's origin and phenotype as inferred by whole-genome sequencing. *Nat. Commun.* **3,** 698 (2012).
7. Olalde, I. *et al.* Derived immune and ancestral pigmentation alleles in a 7,000-year-old Mesolithic European. *Nature* (2014). doi:10.1038/nature12960
8. Skoglund, P. *et al.* Genomic Diversity and Admixture Differs for Stone-Age Scandinavian Foragers and Farmers. *Science* **344,** 747–750 (2014).
9. Milisauskas, S. *European Prehistory: A Survey*. (Springer, 2011).
10. Whittle, A. *Europe in the Neolithic: the Creation of New Worlds*. (Cambridge University Press, 1996).
11. Nandris, J. in *Short Walk Balk. First Farmers Carpathian Basin Adjac. Reg.* (eds. Spataro, M. & Biagi, P.) 11–23 (Società per la Preistoria e Protostoria della Regione Friuli-Venezia Giulia, Quaderno 12, 2007).
12. Anders, A. & Siklósi, Z. in *Br. Archaeol. Rep.* **2334,** (BAR International Series, 2012).
13. Kalicz, N. & Makkay, J. *Die Linienbandkeramik in der Großen Ungarischen*. (Akadémiai Kiadó, 1977).
14. Sherratt, A. *Economy and Society in Prehistoric Europe. Changing Perspectives*. (Edinburgh University Press, 1997).
15. Oross, K. & Bánffy, E. Three successive waves of Neolithisation: LBK development in Transdanubia. *Doc. Praehist.* **36,** 175–189 (2009).
16. McIntosh, J. *Handbook to Life in Prehistoric Europe*. (Oxford University Press, 2009).
17. Lam, Y. M., Chen, X. & Pearson, O. M. Intertaxonomic Variability in Patterns of Bone Density and the Differential Representation of Bovid, Cervid, and Equid Elements in the Archaeological Record. *Am Antiq* **64,** 343–362 (1999).
18. Li, J. Z. *et al.* Worldwide Human Relationships Inferred from Genome-Wide Patterns of Variation. *Science* **319,** 1100–1104 (2008).
19. Behar, D. M. *et al.* The genome-wide structure of the Jewish people. *Nature* **466,** 238–242 (2010).
20. Yunusbayev, B. *et al.* The Caucasus as an Asymmetric Semipermeable Barrier to Ancient Human Migrations. *Mol. Biol. Evol.* **29,** 359–365 (2012).
21. Domboróczki, L., Kaczanowska, M. & Kozłowski, J. The Neolithic settlement at Tiszaszőlős-Domaháza-puszta and the question of the northern spread of the Körös Culture. *Atti Della Soc. Preistoria E Protostoria Della Reg. Friuli-Venezia Giulia* **17,** 101–155 (2010).

22. Bollongino, R. *et al.* 2000 Years of Parallel Societies in Stone Age Central Europe. *Science* 1245049 (2013). doi:10.1126/science.1245049
23. Brandt, G. *et al.* Ancient DNA Reveals Key Stages in the Formation of Central European Mitochondrial Genetic Diversity. *Science* **342,** 257–261 (2013).
24. Haak, W. *et al.* Ancient DNA from the First European Farmers in 7500-Year-Old Neolithic Sites. *Science* **310,** 1016–1018 (2005).
25. Lacan, M. *et al.* Ancient DNA suggests the leading role played by men in the Neolithic dissemination. *Proc. Natl. Acad. Sci.* **108,** 18255 –18259 (2011).
26. Cunliffe, B. *Europe Between the Oceans: 9000 Bc-Ad 1000*. (Yale University Press, 2011).
27. Pasaniuc, B. *et al.* Extremely low-coverage sequencing and imputation increases power for genome-wide association studies. *Nat Genet* **44,** 631–635 (2012).
28. 1000 Genomes Project Consortium *et al.* An integrated map of genetic variation from 1,092 human genomes. *Nature* **491,** 56–65 (2012).
29. Alexander, D. H., Novembre, J. & Lange, K. Fast model-based estimation of ancestry in unrelated individuals. *Genome Res* **19,** 1655–1664 (2009).
30. Pemberton, T. J. *et al.* Genomic Patterns of Homozygosity in Worldwide Human Populations. *Am J Hum Genet* **91,** 275–292 (2012).
31. MacLeod, I. M., Larkin, D. M., Lewin, H. A., Hayes, B. J. & Goddard, M. E. Inferring demography from runs of homozygosity in whole-genome sequence, with correction for sequence errors. *Mol Biol Evol* **30,** 2209–2223 (2013).
32. Wilde, S. *et al.* Direct evidence for positive selection of skin, hair, and eye pigmentation in Europeans during the last 5,000 y. *Proc Natl Acad Sci U A* (2014). doi:10.1073/pnas.1316513111
33. Beleza, S. *et al.* The timing of pigmentation lightening in Europeans. *Mol Biol Evol* **30,** 24–35 (2012).
34. Itan, Y., Powell, A., Beaumont, M. A., Burger, J. & Thomas, M. G. The origins of lactase persistence in Europe. *PLoS Comput Biol* **5,** (2009).
35. Sabeti, P. C. *et al.* Genome-wide detection and characterization of positive selection in human populations. *Nature* **449,** 913–918 (2007).
36. Craig, O. E. (Oliver) *et al.* Did the first farmers of central and eastern Europe produce dairy foods? (2005).
37. Burger, J., Kirchner, M., Bramanti, B., Haak, W. & Thomas, M. G. Absence of the lactase-persistence-associated allele in early Neolithic Europeans. *Proc. Natl. Acad. Sci.* **104,** 3736 –3741 (2007).
38. Sverrisdóttir, O. Ó. *et al.* Direct estimates of natural selection in Iberia indicate calcium absorption was not the only driver of lactase persistence in Europe. *Mol Biol Evol* (2014). doi:10.1093/molbev/msu049
39. McKenna, A. *et al.* The Genome Analysis Toolkit: a MapReduce framework for analyzing next-generation DNA sequencing data. *Genome Res* **20,** 1297–1303 (2010).
40. Browning, S. R. & Browning, B. L. Rapid and accurate haplotype phasing and missing-data inference for whole-genome association studies by use of localized haplotype clustering. *Am J Hum Genet* **81,** 1084–1097 (2007).
41. Jónsson, H., Ginolhac, A., Schubert, M., Johnson, P. L. F. & Orlando, L. mapDamage2.0: fast approximate Bayesian estimates of ancient DNA damage parameters. *Bioinformatics* **29,** 1682–1684 (2013).
42. Skoglund, P., Storå, J., Götherström, A. & Jakobsson, M. Accurate sex identification of ancient human remains using DNA shotgun sequencing. *J. Archaeol. Sci.* **40,** 4477–4482 (2013).

43. Pécsi, M. & Sárfalvi, B. *The Geography of Hungary*. (Corvina Press, 1964).
44. Chapman, J. in *Anc. Bulg. Pap. Present. Int. Symp. Anc. Hist. Archaeol. Bulg. Univ. Nottm. 1981* (ed. Poulter, A.) 1–41 (University of Nottingham, 1983).
45. Lichter, C. l. *Untersuchungen zu den Besattunggssitten des südosteuropäischen Neolithikums und Chalkolithikums*. (Zabern, 2001).
46. Paluch, T. in *First Neolit. Sites Cent.-East Eur. Transect Vol. III Körös Cult. East. Hung.* (eds. Anders, A. & Siklósi, Z.) 179–185 (BAR International Series 2334, 2012).
47. Duffy, P., Parkinson, W., Gyucha, A. & Yerkes, R. W. in *Prehist. Villages Cities Settl. Aggreg. Community Transform.* (ed. Birch, J.) 44–62 (Routledge, 2013).
48. Siklósi, Z. A társadalmi egyenlőtlenség nyomai a késő neolitikumban a Kárpátmedence keleti felén (Traces of social inequality during the Late Neolithic in the Eastern Carpathian Basin). (2013).
49. Horváth, L. A. & Virág, M. zs. in *Hung. Archaeol. Turn Millenn.* (ed. Visy, Z.) 125–127 (Ministry of National Cultural Heritage & Teleki László Foundation, 2003).
50. Parkinson, W. A. *The Social Organization of Early Copper Age Tribes on the Great Hungarian Plain*. (BAR International Series 1573, 2006).
51. Gerling, C. *et al.* Immigration and transhumance in the Early Bronze Age Carpathian Basin: the occupants of a kurgan. *Antiquity* **334,** 1097–1111 (2012).
52. Kemenczei, T. in *Hung. Archaeol. Turn Millenn.* (ed. Visy, Z.) 167–174 (Ministry of National Cultural Heritage & Teleki László Foundation, 2003).
53. Vaday, A. in *Hung. Archaeol. Turn Millenn.* (ed. Visy, Z.) 483–488 (Ministry of National Cultural Heritage & Teleki László Foundation, 2003).
54. Kemenczei, T. *Studien zu den Denkmälern skytisch geprägter Alföld Gruppe*. (Magyar Nemzeti Múzeum, 2009).
55. Domboróczki, L. in *Neolit. Carpathian Basin Northernmost Distrib. StarčevoKörös Cult.* (eds. Kozłowski, J. & Raczky, P.) 137–176 (Polska Akademia Umiejętnoś, 2010).
56. Szathmáry, L. Koraneolitikus (AVK) csontvázleletek Berettyószentmárton-Morotva lelőhelyről. *Debreceni Déri Múz. Évkönyve* 31–34 (1979).
57. Sz. Máthé, M. Újkőkori település Berettyószentmárton-Morotva lelőhelyen. *Debreceni Déri Múz. Évkönyve* **1978,** 35–56 (1979).
58. Paluch, T. The Körös culture graves. *Soc. Preistoria E Protostoria Della Reg. Friuli-Venezia Giulia* **11,** 247–257 (2007).
59. Raczky, P. Polgár, Ferenci-hát. *Régészeti Kut. Magyarországon 2002 Archaeol. Investig. Hung.* 257–258 (2004).
60. Raczky, P. & Anders, A. in *Neolit. Kreisgrabenanlagen Eur. Int. Arbeitstagung 7–9 Mai 2004 Goseck Sachs.-Anhalt* (eds. Bertemes, F., Biehl, P. & Meller, H.) 271–309 (Tagungen des Landesmuseums für Vorgeschichte Halle, 2012).
61. Raczky, P. & Anders, A. in *Interact. Differ. Models Neolit. North Cent. Eur. Agro-Ecol. Barrier Pap. Present. Symp. Organ. EU Proj. FEPRE* (ed. Kozłowski, J.) 31–50 (Prace Komisji Prehistorii Karpat PAU 5, 2009).
62. Whittle, A. *et al.* in *First Farmers Cent. Eur. Divers. LBK Lifeways* (eds. Whittle, A. & Bickle, P.) 49–100 (Oxbow Books, 2013).
63. Dani, J. Preliminary report on the excavation at Debrecen-Nyulas (‘Toyota-Szalon’), Eastern Hungary. *Ősrégészeti Levelek Prehist. Newsl.* **8-9,** 51–53 (2007).
64. Zoffmann, Z. Human remains from the Esztár group site of Debrecen-Nyulas (‘Toyota Szalon’), Eastern Hungary. *Ősrégészeti Levele Prehist. Newsl.* **8-9,** 55–56 (2007).

65. Hajdú, Z. & Nagy, E. G. Debrecen, Tócó-part. *Erdőalja Régészeti Kut. Magyarországon Archaeol. Investig. Hung.* 182–183 (2009).
66. Csengeri, P. Középső neolitikus arcos edények Garadnáról (Hernád-Völgy) – (Middle Neolithic vessels with human face representations from Garadna (Hernád valley, Northeast Hungary). *Herman Ottó Múz. Évkv.* **50,** 67–104 (2011).
67. Csengeri, P. Settlements of the Bükk culture from Hernád valley, north-eastern Hungary. *Archeometriai Műh.* **7,** 227–236 (2010).
68. Domboróczki, L. Radiocarbon Data from Neolithic Archaeological Sites in Heves County (North-Eastern Hungary). *Agria* **38,** 5–71 (2003).
69. Domboróczki, L. Régészeti-kutatások Ludas, Varjú-dűlőn 1998 és 2002 között (Előzetes jelentés) (Archaeological investigations at Ludas, Varjú-dűlő between 1998 and 2002 (Preliminary Report)). *Régészeti Kut. MagyarországonArchaeological Investig. Hung.* 5–23 (2004).
70. Domboróczki, L. in *Nécropole Celt. À Ludas-Varjú-Dűlő* (ed. Szabó, M.) 155–169 (L'Harmattan, 2012).
71. Zoffmann, Z. A preszkíta Mezőcsáti kultúra embertani áttekintése és újabb leletei Ludas–Varjú-dűlő lelőhelyről (Anthropological outline of the pre-scythian Mezőcsát culture and its new finds from the Ludas–Varjú-dűlő site). *Anthr. Közlemények* **49,** 35–42 (2008).
72. Yang, D. Y., Eng, B., Waye, J. S., Dudar, J. C. & Saunders, S. R. Technical note: improved DNA extraction from ancient bones using silica-based spin columns. *Am J Phys Anthr.* **105,** 539–543 (1998).
73. MacHugh, D., Edwards, C., Bailey, J., Bancroft, D. & others. The extraction and analysis of ancient DNA from bone and teeth: a survey of current methodologies. *Anc Biomol* (2000).
74. Orlando, L. *et al.* True single-molecule DNA sequencing of a Pleistocene horse bone. *Genome Res* (2011). doi:10.1101/gr.122747.111
75. Meyer, M. & Kircher, M. Illumina sequencing library preparation for highly multiplexed target capture and sequencing. *Cold Spring Harb Protoc* **2010,** db.prot5448 (2010).
76. Green, R. E. *et al.* A Draft Sequence of the Neandertal Genome. *Science* **328,** 710–722 (2010).
77. Meyer, M. *et al.* A High-Coverage Genome Sequence from an Archaic Denisovan Individual. *Science* **338,** 222–226 (2012).
78. Martin, M. Cutadapt removes adapter sequences from high-throughput sequencing reads. *EMBnet J.* **17,** 10–12 (2011).
79. Briggs, A. W. *et al.* Patterns of damage in genomic DNA sequences from a Neandertal. *Proc. Natl. Acad. Sci.* **104,** 14616–14621 (2007).
80. Brotherton, P. *et al.* Novel high-resolution characterization of ancient DNA reveals C > U-type base modification events as the sole cause of post mortem miscoding lesions. *Nucleic Acids Res* **35,** 5717–5728 (2007).
81. Li, H. & Durbin, R. Fast and accurate short read alignment with Burrows-Wheeler transform. *Bioinformatics* **25,** 1754–1760 (2009).
82. Li, H. *et al.* The Sequence Alignment/Map (SAM) Format and SAMtools. *Bioinformatics* btp352 (2009). doi:10.1093/bioinformatics/btp352
83. Yunusbayev, B. *et al.* The Caucasus as an asymmetric semipermeable barrier to ancient human migrations. *Mol Biol Evol* (2011). doi:10.1093/molbev/msr221
84. Patterson, N., Price, A. L. & Reich, D. Population structure and eigenanalysis. *PLoS Genet* **2,** e190 (2006).

85. Danecek, P. *et al.* The Variant Call Format and VCFtools. *Bioinformatics* (2011). doi:10.1093/bioinformatics/btr330
86. Sánchez-Quinto, F. *et al.* Genomic Affinities of Two 7,000-Year-Old Iberian Hunter-Gatherers. *Curr Biol* **22,** 1494–1499 (2012).
87. Rohland, N., Siedel, H. & Hofreiter, M. A rapid column-based ancient DNA extraction method for increased sample throughput. *Mol Ecol Resour* **10,** 677–683 (2010).
88. Ginolhac, A., Rasmussen, M., Gilbert, M. T. P., Willerslev, E. & Orlando, L. mapDamage: testing for damage patterns in ancient DNA sequences. *Bioinformatics* **27,** 2153–2155 (2011).
89. Shapiro, B. & Hofreiter, M. A Paleogenomic Perspective on Evolution and Gene Function: New Insights from Ancient DNA. *Science* **343,** (2014).
90. Sawyer, S., Krause, J., Guschanski, K., Savolainen, V. & Pääbo, S. Temporal Patterns of Nucleotide Misincorporations and DNA Fragmentation in Ancient DNA. *PLoS One* **7,** e34131 (2012).
91. Rasmussen, M. *et al.* An Aboriginal Australian Genome Reveals Separate Human Dispersals into Asia. *Science* **334,** 94–98 (2011).
92. Gilbert, M., Shapiro, B., Drummond, A. & Cooper, A. Post-mortem DNA damage hotspots in Bison (Bison bison) provide evidence for both damage and mutational hotspots in human mitochondrial DNA. *J Archaeol Sci* **32,** 1053–1060 (2005).
93. Wall, J. D. & Kim, S. K. Inconsistencies in Neanderthal genomic DNA sequences. *PLoS Genet* **3,** 1862–1866 (2007).
94. Zhidkov, I., Nagar, T., Mishmar, D. & Rubin, E. MitoBamAnnotator: A web-based tool for detecting and annotating heteroplasmy in human mitochondrial DNA sequences. *Mitochondrion* **11,** 924–928 (2011).
95. Kloss-Brandstätter, A. *et al.* HaploGrep: a fast and reliable algorithm for automatic classification of mitochondrial DNA haplogroups. *Hum Mutat* **32,** 25–32 (2011).
96. Van Oven, M. & Kayser, M. Updated comprehensive phylogenetic tree of global human mitochondrial DNA variation. *Hum Mutat* **30,** E386–E394 (2009).
97. Behar, D. M. *et al.* A 'Copernican' Reassessment of the Human Mitochondrial DNA Tree from its Root. *Am J Hum Genet* **90,** 675–684 (2012).
98. Fedorova, S. A. *et al.* Autosomal and uniparental portraits of the native populations of Sakha (Yakutia): implications for the peopling of Northeast Eurasia. *BMC Evol Biol* **13,** 127 (2013).
99. Richards, M. *et al.* Tracing European founder lineages in the Near Eastern mtDNA pool. *Am J Hum Genet* **67,** 1251–1276 (2000).
100. Macaulay, V. *et al.* The emerging tree of West Eurasian mtDNAs: a synthesis of control-region sequences and RFLPs. *Am J Hum Genet* **64,** 232–249 (1999).
101. Behar, D. M. *et al.* MtDNA evidence for a genetic bottleneck in the early history of the Ashkenazi Jewish population. *Eur J Hum Genet* **12,** 355–364 (2004).
102. Lacan, M. *et al.* Ancient DNA suggests the leading role played by men in the Neolithic dissemination. *Proc. Natl. Acad. Sci.* 105–114 (2011). doi:10.1073/pnas.1113061108
103. Hervella, M. *et al.* Ancient DNA from Hunter-Gatherer and Farmer Groups from Northern Spain Supports a Random Dispersion Model for the Neolithic Expansion into Europe. *PLoS One* **7,** e34417 (2012).
104. Haak, W. *et al.* Ancient DNA from European early neolithic farmers reveals their near eastern affinities. *PLoS Biol* **8,** e1000536 (2010).

105. Gamba, C. *et al.* Ancient DNA from an Early Neolithic Iberian population supports a pioneer colonization by first farmers. *Mol Ecol* **21,** 45–56 (2012).
106. Lacan, M. *et al.* Ancient DNA reveals male diffusion through the Neolithic Mediterranean route. *Proc Natl Acad Sci U A* **108,** 9788–9791 (2011).
107. García, O. *et al.* Using mitochondrial DNA to test the hypothesis of a European post-glacial human recolonization from the Franco-Cantabrian refuge. *Heredity* **106,** 37–45 (2011).
108. Tambets, K. *et al.* The Western and Eastern Roots of the Saami—the Story of Genetic 'Outliers' Told by Mitochondrial DNA and Y Chromosomes. *Am J Hum Genet* **74,** 661–682 (2004).
109. Malyarchuk, B. *et al.* The Peopling of Europe from the Mitochondrial Haplogroup U5 Perspective. *PLoS One* **5,** e10285 (2010).
110. Bramanti, B. *et al.* Genetic Discontinuity Between Local Hunter-Gatherers and Central Europe's First Farmers. *Science* **326,** 137–140 (2009).
111. Malmström, H. *et al.* Ancient DNA Reveals Lack of Continuity between Neolithic Hunter-Gatherers and Contemporary Scandinavians. *Curr Biol* **19,** 1758–1762 (2009).
112. Brotherton, P. *et al.* Neolithic mitochondrial haplogroup H genomes and the genetic origins of Europeans. *Nat Commun* **4,** 1764 (2013).
113. Reidla, M. *et al.* Origin and Diffusion of mtDNA Haplogroup X. *Am J Hum Genet* **73,** 1178–1190 (2003).
114. Deguilloux, M.-F. *et al.* News from the west: Ancient DNA from a French megalithic burial chamber. *Am J Phys Anthr.* **144,** 108–118 (2011).
115. Haak, W. *et al.* Ancient DNA, Strontium isotopes, and osteological analyses shed light on social and kinship organization of the Later Stone Age. **105,** 18226–18231 (2008).
116. Pala, M. *et al.* Mitochondrial DNA signals of late glacial recolonization of Europe from near eastern refugia. *Am J Hum Genet* **90,** 915–924 (2012).
117. Sampietro, M. *et al.* Palaeogenetic evidence supports a dual model of Neolithic spreading into Europe. *Proc R Soc B* **274,** 2161–2167 (2007).
118. Palanichamy, M. *et al.* Mitochondrial haplogroup N1a phylogeography, with implication to the origin of European farmers. *BMC Evol Biol* **10,** 304 (2010).
119. Tanaka, M. *et al.* Mitochondrial genome variation in eastern Asia and the peopling of Japan. *Genome Res* **14,** 1832–1850 (2004).
120. Kemenczei, T. in *Hung. Archaeol. Turn Millenn.* (ed. Visy, Z.) 177–179 (Budapest: Ministry of National Cultural Heritage, Teleki László Foundation, 2003).
121. Rootsi, S. *et al.* Phylogeography of Y-chromosome haplogroup I reveals distinct domains of prehistoric gene flow in europe. *Am J Hum Genet* **75,** 128–137 (2004).
122. Soares, P. *et al.* The archaeogenetics of Europe. *Curr Biol* **20,** R174–83 (2010).
123. Francalacci, P. *et al.* Low-pass DNA sequencing of 1200 Sardinians reconstructs European Y-chromosome phylogeny. *Science* **341,** 565–569 (2013).
124. Zhong, H. *et al.* Global distribution of Y-chromosome haplogroup C reveals the prehistoric migration routes of African exodus and early settlement in East Asia. *J Hum Genet* **55,** 428–435 (2010).
125. Scozzari, R. *et al.* Molecular Dissection of the Basal Clades in the Human Y Chromosome Phylogenetic Tree. *PLoS One* **7,** e49170 (2012).

126. Semino, O., Passarino, G., Brega, A., Fellous, M. & Santachiara-Benerecetti, A. S. A view of the neolithic demic diffusion in Europe through two Y chromosome-specific markers. *Am J Hum Genet* **59,** 964–968 (1996).
127. Hammer, M. F. *et al.* Out of Africa and back again: nested cladistic analysis of human Y chromosome variation. *Mol Biol Evol* **15,** 427–441 (1998).
128. Rootsi, S. *et al.* Distinguishing the co-ancestries of haplogroup G Y-chromosomes in the populations of Europe and the Caucasus. *Eur J Hum Genet* **20,** 1275–1282 (2012).
129. Di Giacomo, F. *et al.* Y chromosomal haplogroup J as a signature of the post-neolithic colonization of Europe. *Hum Genet* **115,** 357–371 (2004).
130. King, R. J. *et al.* Differential Y-chromosome Anatolian Influences on the Greek and Cretan Neolithic. *Ann Hum Genet* **72,** 205–214 (2008).
131. Cinnioğlu, C. *et al.* Excavating Y-chromosome haplotype strata in Anatolia. *Hum Genet* **114,** 127–148 (2003).
132. Karafet, T. *et al.* Paternal population history of East Asia: sources, patterns, and microevolutionary processes. *Am J Hum Genet* **69,** 615–628 (2001).
133. Rootsi, S. *et al.* A counter-clockwise northern route of the Y-chromosome haplogroup N from Southeast Asia towards Europe. *Eur J Hum Genet* **15,** 204–211 (2006).
134. Core Team, R. R: A Language and Environment for Statistical Computing. at <http://www.R-project.org/>
135. Oksanen, J. *et al.* vegan: Community Ecology Package. (2013). at <http://CRAN.R-project.org/package=vegan>
136. Purcell, S. *et al.* PLINK: a tool set for whole-genome association and population-based linkage analyses. *Am J Hum Genet* **81,** 559–575 (2007).
137. McQuillan, R. *et al.* Runs of homozygosity in European populations. *Am J Hum Genet* **83,** 359–372 (2008).
138. Kirin, M. *et al.* Genomic Runs of Homozygosity Record Population History and Consanguinity. *PLoS One* **5,** e13996 (2010).
139. Nalls, M. A. *et al.* Measures of Autozygosity in Decline: Globalization, Urbanization, and Its Implications for Medical Genetics. *PLoS Genet* **5,** e1000415 (2009).
140. Skotte, L., Korneliussen, T. S. & Albrechtsen, A. Estimating individual admixture proportions from next generation sequencing data. *Genetics* **195,** 693–702 (2013).
141. Relethford, J. H. Hemispheric difference in human skin color. *Am J Phys Anthr.* **104,** 449–457 (1997).
142. Parra, E. J. Human pigmentation variation: evolution, genetic basis, and implications for public health. *Am J Phys Anthr.* **Suppl 45,** 85–105 (2007).
143. Sturm, R. A. Molecular genetics of human pigmentation diversity. *Hum Mol Genet* **18,** R9–17 (2009).
144. Rees, J. L. & Harding, R. M. Understanding the evolution of human pigmentation: recent contributions from population genetics. *J Invest Dermatol* **132,** 846–853 (2011).
145. Norton, H. L. *et al.* Genetic Evidence for the Convergent Evolution of Light Skin in Europeans and East Asians. *Mol Biol Evol* **24,** 710–722 (2006).
146. Pickrell, J. K. *et al.* Signals of recent positive selection in a worldwide sample of human populations. *Genome Res* **19,** 826–837 (2009).
147. Lao, O., de Gruijter, J. M., van Duijn, K., Navarro, A. & Kayser, M. Signatures of positive selection in genes associated with human skin pigmentation as

revealed from analyses of single nucleotide polymorphisms. *Ann Hum Genet* **71,** 354–369 (2007).
148. Myles, S., Somel, M., Tang, K., Kelso, J. & Stoneking, M. Identifying genes underlying skin pigmentation differences among human populations. *Hum Genet* **120,** 613–621 (2006).
149. Ingram, C. J. E., Mulcare, C. A., Itan, Y., Thomas, M. G. & Swallow, D. M. Lactose digestion and the evolutionary genetics of lactase persistence. *Hum Genet* **124,** 579–591 (2008).
150. Itan, Y., Jones, B. L., Ingram, C. J. E., Swallow, D. M. & Thomas, M. G. A worldwide correlation of lactase persistence phenotype and genotypes. *BMC Evol Biol* **10,** 36 (2010).
151. Bersaglieri, T. *et al.* Genetic Signatures of Strong Recent Positive Selection at the Lactase Gene. *Am J Hum Genet* **74,** 1111–1120 (2004).
152. Enattah, N. S. *et al.* Identification of a variant associated with adult-type hypolactasia. *Nat Genet* **30,** 233–237 (2002).
153. Burger, J., Kirchner, M., Bramanti, B., Haak, W. & Thomas, M. G. Absence of the lactase-persistence-associated allele in early Neolithic Europeans. *Proc. Natl. Acad. Sci.* **104,** 3736–3741 (2007).
154. Malmström, H. *et al.* High frequency of lactose intolerance in a prehistoric hunter-gatherer population in northern Europe. *BMC Evol Biol* **10,** 89 (2010).
155. Pneuman, A., Budimlija, Z. M., Caragine, T., Prinz, M. & Wurmbach, E. Verification of eye and skin color predictors in various populations. *Leg Med* **14,** 78–83 (2012).
156. Spichenok, O. *et al.* Prediction of eye and skin color in diverse populations using seven SNPs. *Forensic Sci Int Genet* **5,** 472–478 (2010).
157. Walsh, S. *et al.* The HIrisPlex system for simultaneous prediction of hair and eye colour from DNA. *Forensic Sci Int Genet* **7,** 98–115 (2012).
